# Crossed and Linked Histories of Tetrapyrrolic Macrocycles and Their Use for Engineering Pores within Sol-Gel Matrices

**DOI:** 10.3390/molecules18010588

**Published:** 2013-01-04

**Authors:** Miguel A. García-Sánchez, Fernando Rojas-González, E. Carmina Menchaca-Campos, Salvador R. Tello-Solís, R. Iris Y. Quiroz-Segoviano, Luis A. Diaz-Alejo, Eduardo Salas-Bañales, Antonio Campero

**Affiliations:** 1 Departamento de Quimica, Universidad Autónoma Metropolitana-Iztapalapa, Av. San Rafael Atlixco 186, Vicentina, D. F. 09340, Mexico; 2 Centro de Investigación en Ingeniería y Ciencias Aplicadas, UAEM, Av. Universidad 1001, Col. Chamilpa, C.P. 62209, Cuernavaca Mor., Mexico

**Keywords:** tetrapyrrole macrocycles, porphyrin, phthalocyanine, sol-gel, silica, alkoxide, pore size, specific surface

## Abstract

The crossed and linked histories of tetrapyrrolic macrocycles, interwoven with new research discoveries, suggest that Nature has found in these structures a way to ensure the continuity of life. For diverse applications porphyrins or phthalocyanines must be trapped inside solid networks, but due to their nature, these compounds cannot be introduced by thermal diffusion; the sol-gel method makes possible this insertion through a soft chemical process. The methodologies for trapping or bonding macrocycles inside pristine or organo-modified silica or inside ZrO_2_ xerogels were developed by using phthalocyanines and porphyrins as molecular probes. The sizes of the pores formed depend on the structure, the cation nature, and the identities and positions of peripheral substituents of the macrocycle. The interactions of the macrocyclic molecule and surface Si-OH groups inhibit the efficient displaying of the macrocycle properties and to avoid this undesirable event, strategies such as situating the macrocycle far from the pore walls or to exchange the Si-OH species by alkyl or aryl groups have been proposed. Spectroscopic properties are better preserved when long unions are established between the macrocycle and the pore walls, or when oligomeric macrocyclic species are trapped inside each pore. When macrocycles are trapped inside organo-modified silica, their properties result similar to those displayed in solution and their intensities depend on the length of the alkyl chain attached to the matrix. These results support the prospect of tuning up the pore size, surface area, and polarity inside the pore cavities in order to prepare efficient catalytic, optical, sensoring, and medical systems. The most important feature is that research would confirm again that tetrapyrrolic macrocycles can help in the development of the authentic pore engineering in materials science.

## Nomenclature

Acac: acetylacetone (CH_3_COCH_2_COCH_3_)
APTES: aminopropyltri-ethoxysilane
AmTEOS: amyltriethoxysilane (C_5_H_1_1)Si(OC_2_H_5_)_3_
AlyTEOS: allyl-triethoxysilane (CH_2_=CH-CH_2_-Si(OC_2_H_5_)_3_)
APDT: antimicrobial photodynamic therapy
B: Soret band
BJH: Barrett, Joyner and Halenda pore-size distribution method
dMeDEOS: dimethyldiethoxysilane, (CH_3_)_2_Si(OC_2_H_5_)_2_
DCB: dicyanobenzene
DMF: dimethylformamide
DoTEOS: dodecyltriethoxysilane (C_1_2H_2_5-Si(OC_2_H_5_)_3_)
EtTEOS: ethyltriethoxysilane (C_2_H_5_)Si(OC_2_H_5_)_3_
FA: functionalized alkoxide
h: η_H_2_O_/η_alkoxy_ (water-alkoxide molar ratio)
Hexa: 1,6-hexanodiamine
HpD: hematoporphyrin derivative
HLn(Pc)_2_: lanthanide bisphthalocyanine
H_2_P: free base of porphyrin
H_2_Pc: free phthalocyanine
HTES: hydride-triethoxysilane (HSi(OC_2_H_5_)_3_)
H_2_TPP: tetra-phenylporphyrin free base
H_2_T(*o* or *p*-S)PP: *ortho (X)* or *para (Y)* substituted tetraphenyl-porphyrins
IPTES: isocyanatopropyltriethoxysilane
Ln(TPP)Ac·2S: lanthanide acetate-tetraphenylporphyrin
Ln(Ac)•nH_2_O: lanthanide acetate
HLn(Pc)_2_: lanthanide bisphthalocyanine
MCM-41, 48, 50, *etc*.: Mobil Corporation Material 41, 48, 50, *etc*.
MeTES: methyl-triethoxysilane (CH_3_Si(OC_2_H_5_)_3_)
MSNP: mesoporous silica nanoparticle
MTSPc: metal tetrasulfophthalocyanine
NAEPTES: *N*-(2-aminoethyl-amino)propyltrimethoxysilane
NHSG: non-hydrolytic sol gel
NIR: Near Infrared Spectroscopy
NMR: Nuclear Magnetic Resonance Spectroscopy
NLDFT: Non-Local Density Functional Theory
(OH)AlTSPc: aluminum hydroxyl-tetrasulfophathalocyanine
OSA: organo-substituted alkoxide
PBG: porphobilinogen
PDT: photodynamic therapy
PMMA: polymethylmetacrylate
PMO’s: Periodic MesoporousOrgano-Silicas
Py: pyridine
Q_I, II, III or IV_: Q bands of tetrapyrrolic macrocycles
R: alkyl or aryl group
r_Ln_: lanthanide ionic radious
S: solvent molecule
SBA-n: Santa Barbara “n” Material
SBA-15: Santa Barbara Material “15”
SEM: Scanning Electron Microscopy
TEOS: tetraethoxysilane (Si(OC_2_H_5_)_4_)
TMOS: tetramethoxysilane (Si(OCH_3_)_4_)
UV-Vis: Ultrviolet and Visible Spectroscopy
V_f_: total volume of gelling mixture
V_TEOS_: tetraethoxysilane volume
VyTEOS: vynil-triethoxysilane (CH_2_=CH-)Si(OC_2_H_5_)_3_
Zr(O^n^Pr)_4_: zirconium n-propoxide (Zr(OCH_2_CH_2_CH_3_)_4_
[H_2_P or MP]n: oligomeric free- or metallo-porphyrin
Ф: average pore diameter
λ: wavelength
λex: fluorescence excitation wavelength

## 1. Crossed and Linked Histories of Tetrapyrrolic or How Nature Allows for No Mistakes

While men have always associated chlorophyll with life in green plants even while recognizing that in other living beings blood is red, the structural similarities of these two substances involved either in the growth of plants or in the human respiratory process, remain mostly ignored. Porphyria*,* a severe genetic disorder that causes psychiatric disturbances among other things, has been responsible of legends about vampires, werewolves and others creatures. However, it was Hippocrates (460–370 BC), who gave the first description of many of the features which now could be associated with an acute attack of porphyria, in a woman from Thasus. At present, it is known that porhyria occurs when any one of the enzymes needed for the production of the heme species results abnormal; then, the process is disturbed and intermediate products, such as porphyrins or its precursors such as porphobilinogen (PBG), can build up and be excreted in urine and stool. This disorder induces a decreased production of the heme group of blood, and concomitant amounts of its precursors. The hepatic porphyria primarily affects the nervous system causing paranoia, depression, and acute cardiac disorders. In severe cases, porphyria species are responsible for strong deformations in the aspect of the patients. The term porphyrin made allusion to the deep purple or violet color observed in many of these compounds; this is so because the Greek porphyra (or the Latin porphurá), means purple pigment. Along the time, histories related to fundamental molecules and linked to the existence of life in earth have been developed in parallel, but the elucidation of the central structures of chlorophyll, hemoglobin, cytochrome or Vitamin B_12_, was only started from the middle of the nineteenth century. Although this manuscript is not written with respect to *porphyria,* this story proceeds in parallel, by coinciding at diverse époques [1], with the stories of chlorophyll, hemoglobin, and cytochromes.

### 1.1. Hemoglobin and Chlorophyll

The name chlorophyll (from the Greek cloros = green and phyllon = leaf) was first coined in 1812 by Pelletier and Caventou to describe the green pigment of the leaves of plants [2,3,4] and, in 1844 Verdeil transformed a green extract of chlorophyll into a red pigment. This observation induced him to suggest the relation existing between this substance and the red blood pigment [3]. In parallel, around 1840 Hunefeld accidentally observed, for the first time, in a dried sample of preserved menstrual blood, crystals of the oxygen carrying protein [4]. In 1841, Scherer obtained an iron-free precipitate from blood when he treated it with concentrated sulfuric acid [5]. In 1844, Mulder described a red-purple compound obtained from blood, but having no iron and branded it as *Eisenfreises hamatin* or iron-free haematin [6]. In 1867 Thudichum called this red substance cruentine and described the spectrum and the splendid blood-red fluorescence of that substance [7].

However, Hoppe-Seyler was the first to appropriately describe the absorption spectrum of the oxygenated red blood pigment, in 1862 [8]. Around 1864, the same prominent researcher called the crystallized protein obtained from blood hematoglobulin or hemoglobin [9] and, subsequently described its reversible oxygenation capacity [10]. In 1871, In turn, Hoppe-Seyler found that iron free haematin really consisted of two substances and named hamatoporphryin to the main of both [11]. Finally, in 1879, Hoppe-Seyler showed the structural similarities between chlorophyll and hemoglobin (hematin) derivatives [12], which confirmed the Verdeil observation.

In 1853 Teichmann observed under the microscope that the blood pigment could be separated into a pigment and a protein when a sample was treated with glacial acetic acid [13]; however, it was Schalfejef in 1885 [14] who introduced the classical separation method of the Teichmann’s Crystals, or *hemin*, by the slow addition of blood into glacial acetic acid saturated with sodium chloride at 100 °C. Later, alternative methods were developed by Necki and Zaleski [15]; nevertheless, Mörner developed an effective method for the separation of esters from the blood pigment or haemin, by treating blood with alcoholic sulfuric acid.

In 1883 MacMunn, observed for the first time a sustance that he called a respiratory pigment (i.e., miohematin or hystohematin) obtained from pigments of a large number of different types of organisms, as those arising from pigeon-breast muscle [16]. What was really obtained by this researcher was a denatured-protein haemochromogen out of haemoglobin, and Hoppe-Seyer criticized the work of MacMunn, which was forgotten for a long time until Kaillin rediscovered these cytochromes in 1926 [17]. The respiratory MacMunn pigments have the same main structure of the pigment obtained from blood. In 1912, Piloty [18] and Küster [19,20], based on degradation experiments, proposed the current structure of haemin. In 1880, Hoppe-Seyler informed over the spectral similarities found between the acid degradation product of chlorophyll and hematoporphyrin [11,21]. In 1883, Soret observed an intense UV-Vis band at around 400 nm in hemoglobin samples [22], which was also observed later by Gamgee in porphyrin solutions [23]. The similarities pointed out by Hoppe-Seyler induced some researchers to suggest that chlorophyll contained iron too [21]. In 1906, Willstätter started the elucidation of the structure of the plant pigment chlorophyll and, in 1912; he showed the existence of chlorophylls a and b and in 1913 identified chlorophyll as an insoluble magnesium compound [24,25,26]. Afterward, in 1915, he was awarded the Nobel Prize “for his research on plant pigments, especially chlorophyll”. In 1929, Fischer synthesized heamin and confirmed the structure proposed by Piloty and Küster so he also received the Nobel Prize in Chemistry in 1930 “for his researches into the constitution of haemin and chlorophyll and especially for his synthesis of haemin” [27,28,29]. Fischer work was mostly centred on the investigation of blood pigments in bile and chlorophyll, while the pyrroles arising from these substances could then be synthesized [30,31,32,33,34]. In this way, the laboratory preparation of heamin represented a long aimed goal for the comprehension of one of the principal life supporting substances. Heamin is the chloride compound of the heme group containing an oxidized iron atom. In 1959, Perutz [35,36,37] determined the molecular structure of hemoglobin by comparing the X-ray diffraction patterns of protein crystals having no heavy atoms attached. During the same time, Kendrew determined the structure of myoglobyn [38,39] and shared with Perutz the Nobel Prize in Chemistry of 1962 “for their studies of structure of globular proteins”. Fischer determined the structure of chlorophyll in 1940 through a degradation procedure and it was only until 1960 that Woodward and Stell achieved the overall synthesis of chlorophyll [40,41]. Woodward earned the Nobel Prize in 1965 for having achieved the total synthesis of complex organic molecules such as chlorophyll.

**Figure 1:**
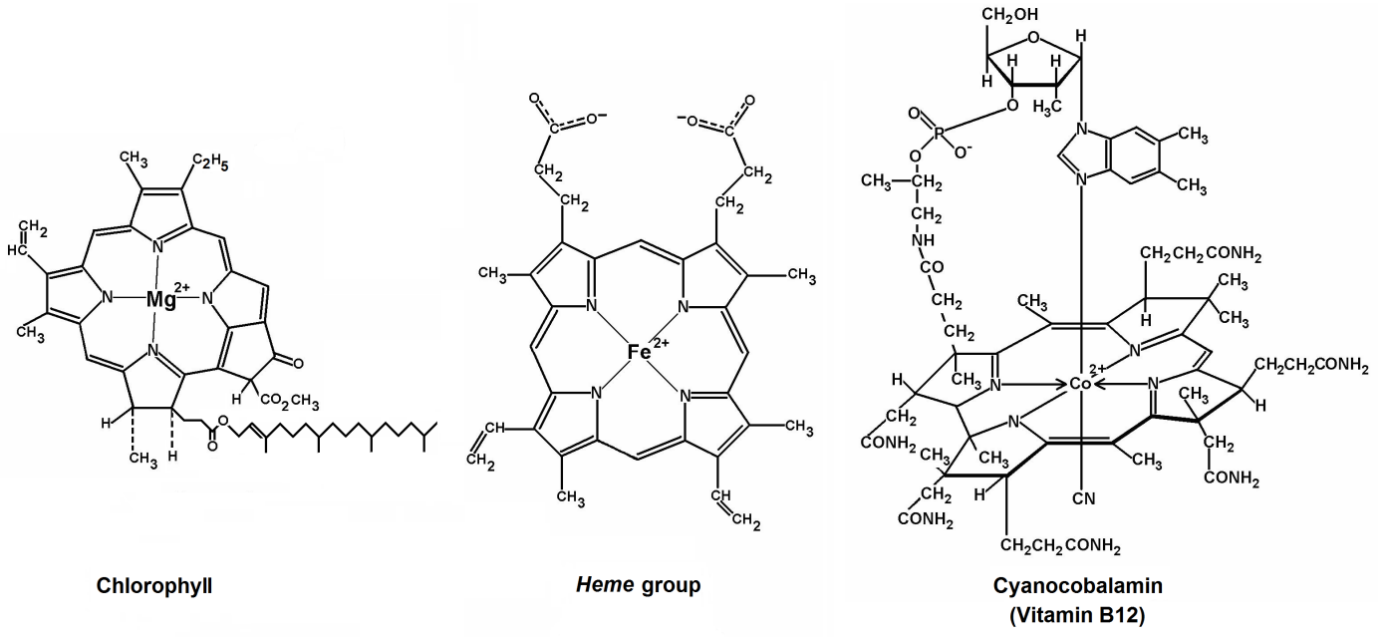
Porphyrinic structures are present in substances playing transcendental roles in life, such as *chlorophyll*, the *heme* group in blood and cytochromes, as well as in other substances as for instance cyanocobalamin.

Fischer and colleges found that tetrapyrrolic macrocycles, in the form of porphyrins and its precursors the pyrroles, are the key for the laboratory synthesis of several vital substances [33]. Pyrrole pigments such as bile pigments, chlorophyll, prodigiosin, vitamin B_12_, and porphyrins are the most abundant natural coloring species. Furthermore, porphyrins are present in the structures of substances (Figure 1) that are essential for the synthesis and regulation of DNA, blood cell formation, and fatty acid synthesis, as for instance cyanocobalamin or vitamin B_12_ [42,43]. A copper porphyrin (i.e., uroporphyrin III) has been found in the feathers of birds, such as the Turacus indicus [44,45]. Due to their natural participation in animals and plants, porphyrins of nickel or vanadium were found in oil shale, bituminous sands, asphalt, and petroleum as consequence of the long time degradation of plants trapped under deep earth layers [46].

There exist an impressive variety of *chlorophylls*, the most abundant ones being chlorophyll a and b, which constitute plentiful photosynthetic pigments in plants. Chlorophyll c is present in the photosynthetic pigments of marine species and a combination of *chlorophyll* d and a, existing in both, Rhodophyceae and green sulfur bacteria [42,47,48]. Similarly, a mixture of chlorophyll a and b was found in the purple pigment of Thiorhodaceae [42]. In order to achieve the energy collection of sunlight and to employ it to transform water and CO2 into usable organic substances during the photosynthesis process, the magnesium porphyrinic complex that is present in *chlorophylls* requires to be endowed with a specific special disposition in photosystems I and II of the plant chloroplast. For this reason, the two *chlorophylls* responsible of the first step of the process, *i.e*., sunlight caption, was called a “special pair”. Additionally, as it is well known, this special pair requires being close of the adequate physicochemical environment as well as in the proximity of another species, such as quinines and cytochromes, in order to perform the required electron transference [47,48,49,50,51,52].

Similarly, the caption, transport, and liberation of O_2_ or CO2 along the respiratory system, is the principal function of the heme B group present in the structures of hemoglobin and myoglobin. This heme group consists of an iron (II) complex of protoporphyrin-IX or Protoheme [42] immersed in a convenient physicochemical environment as for instance the flexible peptide chains that surround the central macrocycle iron atom. In the respiratory process of aerobic organisms, the transport and storage of O_2_ are responsible of the tetrameric hemo structure of hemoglobin and of the monomeric unit of hemo in myoglobin, respectively. In hemoglobin or myoglobin, the hemo species are enveloped by a polypeptide chain, existing in each hemo group and an iron (II) atom pentacoordinated to four nitrogens in the porphyrinic macrocycle and to imidazole in the histidine segment belonging to the protein chain. Through this spatial configuration, iron (II) can coordinate diverse species at its six bonding positions, in an axial and reversible form, such as O2, CO2, *etc*. In hemoglobin, the adjacent peptide chains create a particular physicochemical environment around the central iron (II) atom while tuning its affinity toward for species such as O2, CO2, NO, CO, *etc*. [37,53] and inhibiting the possibility of its irreversible oxidation and possible μ-oxo dimer formation [54,55,56]. As Pauling proposed in 1964 [57], and it was later proved [58,59,60,61,62], a second imidazole molecule due to another histidine residue can interact via H-bonding with the terminal oxygen atom.

### 1.2. The Cytochromes

The heme A or heme B groups that are present in cytochromes [63,64,65,66,67,68,69,70] perform crucial functions in metabolic processes, such as in electron transport [54,63] in oxidative phosphorylation [71], ATP synthesis [67,68], proton pumping [68,72,73] or translocation of protons, promotion of cellular respiration [66,72,73], and oxidation of organic substances [63]. The cytochromes are porphyrinic hematoproteins bonded to the cell membrane, which exist as monomeric proteins (cytochrome c) or subunits of enzymatic compounds and participate in diverse metabolic processes. In most of these latter structures, the porphyrinic type structures require to be endowed with the adequate spatial disposition together with and under the appropriate physicochemical conditions for a correct performance [65,66,67,68,69]. As it was mentioned above, cytochromes were initially described by MacMunn in 1884 [16] as respiratory pigments, but then again this discovery was rapidly overseen until it was rediscovered 40 year later when Keilin defined them as cellular pigments [17] or cytochromes. The name arises from the terms *cyto* (cell) and *chromo* (color), and Keilin classified them by the position of their lower energy band in UV-Vis spectra as cytochrome *a* (605 nm), *b* (~565 nm) and *c* (550 nm).

### 1.3. Cyanocobalamin

The fourth parallel story is concerned with vitamin B_12_ which is a cobalt corrin complex; a parent or derived porphyrinoid-type macrocycle, produced by microorganisms living in symbiosis on the stems of some plants and found in foods that proceed from animals, which include fish, birds, meat (especially liver), poultry, eggs, milk, and its derivatives, as well as in some red and green algae (Nori), chlorella, and aloe vera [74,75]. This substance is essential for blood cell formation, a proper functioning of the brain and nervous system; the synthesis and regulation of DNA, fatty acid synthesis, and energy production [74].

Combe in 1824 was the first to describe [76] a case of severe pallor illness in a man; and the clinical pathological description of Addison [77] permitted to recognize it as a disease which was first referred as pernicious anaemia by Biermer [78], in view of the insidious course of the disease. It was only when recognition of the liver participation in haematopoiesis that the treatment of pernicious anaemia was recognized as empiric and futile. After the First World War, Whipple, who had interest in liver diseases, investigated the function of liver in haematopoiesis, to evaluate the effects of various treatments for curing acute anaemia in dogs and began assessing the effects of some treatments for anaemia caused by chronic blood loss. Together with Hooper and Robscheit-Robbins, Whipple studied the effects on haemoglobin and blood regeneration through diverse treatments [79,80,81,82].

Fortune crossed in front of the eyes of Whipple when he observed poor blood regeneration in dogs fed with cooked liver, whilst a better recuperation took place in dogs nourished with uncooked liver by the indolent laboratory technician.

While visiting and learning from Whipple’s discovery, Minot and Murphy [83,84], decided to try a raw liver supply as a possible treatment for pernicious anemia then finding that a daily diet caused a rapid symptomatic improvement, concomitant with a red cell concentration elevation. In their published results of 1926 [83,84], Minot and Murphy described their previous attempts to treat the illness through such a diet; in particular mentioning Whipple’s work. Consequently Minot and Murphy together with Whipple were awarded the Nobel Prize in 1934. The understanding of pernicious anaemia increased with time and it is now known that the disease is associated with defects in the gastro-intestinal tract that causes the patient to suffer chronic gastritis and a lack of acid secretion. The transport of vitamin B_12_ depends on the combined actions of gastric juices, ileal, and pancreatic components. Castle discovered in 1930 that the stomach of good health patients secreted a substance, or intrinsic factor, which promoted an effective absorption, a process which was absent from the stomachs of patients with pernicious anaemia [85]. These observation induced Castle to administer pre-digested meat or liver aspirated from the stomachs of normal subjects as a possible treatment against of pernicious anemia. In the early 1960s, pernicious anaemia was recognized as an autoimmune disease [86]. Due to the fact that stomach cannot easily digests crude liver, injectable intramuscular extracts of liver were developed in the 1950s for the standard management of pernicious anaemia. Until 1948 the anti-pernicious anaemia factor could be isolated from liver and kidney By Smith [87] and Rickes [88], who called it Vitamin B_12_. These researchers showed that the administration of a few micrograms of this vitamin could prevent the accentuation of the disease. The X-ray crystal structure of Vitamin B_12_ or cyanocobalamin was determined by Hodking in 1956; a work for which she was awarded the Nobel Prize in Medicine in 1963 [89]. Until 1960, Friedrich *et al*. [90] reported a partial synthesis of cyanocobalamine; it was only in 1973 that Woodward [91,92] and Eschenmoser [93,94] reported the first method to synthesize vitamin B_12_ in documents considered classic papers in this field of research.

While the above intricate stories reflect the importance of tetrapyrrolic macrocycles in living structures, it is clear that these species requires of being immersed in the adequate physicochemical environment for a correct functioning. The structural similarities of chlorophyll, the heme group in hemoglobin, and cytochromes as well as those of the porphyrinoid structure in cyanocobalamin are not coincidences, since through these structures sunlight is converted into chemical energy and electrons or O_2_ and CO2 can be captured and transported, and used by animals and plants. The beauty revealed by the comprehension of the role played by tetrapyrrolic species in photosynthesis, respiratory and metabolic processes demonstrate that Nature makes no mistakes and has found in these structures an elegant way to warrant an effective life support.

### 1.4. Tetrapyrrole Macrocycles and Photodynamic Therapy (PDT)

The technique known as photodynamic therapy (PDT) [95,96,97] is based on the selective absorption of some substances called photosensitizers (such as the tetrapyrrolic macrocycles that include porphyrins, chlorins, purpurins, texaphyrins, benzoporphyrins, naphthalocyanines, and phthalocyanines) localized in pathological tissues, as for instance, cancerous cells, and exterminate them when the region is irradiated with light. UV or visible laser light, principally red [95,96,97], that causes the photosensitizer to attain a triplet state, or to render a biological radical substrate that appears after its interaction with it, to react with oxygen in order to create the highly cytotoxic singlet oxygen (^1^O_2_), thus causing the death of malignant cells. Current theories about the mechanisms of PDT principally concern active species that involve oxygen, especially singlet oxygen and the hydroxyl radical, OH^•−^ [98,99,100]. The sharpest absorption peaks of clinical photosensitizers are found in the blue and UV regions of the spectral range; in this part of the spectrum, radiation attains poor tissue penetration. Maximum effects are instead observed with light in the wavelength range between 620 and 690 nm at which optical radiation penetrates about 1.0 cm into biological tissues [101,102,103]. However, at both the far red or near infrared radiation regions (650 to 1,500 nm), a better tissue penetration occurs [101,102,103,104,105] and many commercial lasers operate in this zone. For this reason, the photosensitizers are normally activated with light of around 630 nm, where most drugs show small absorption peaks, but for which many tissues show reasonable penetration. To obtain light of this wavelength, gold vapour lasers (630 nm) or tunable dye lasers are employed; these types of lasers are usually coupled to an optical fibre delivery system.

It is also well known that many porphyrins exhibit a red fluorescence in the range extending from 600 to 730 nm [42,43,106,107,108]. To be considered an efficient PDT application, it is necessary to synthesize photosensitizers that can effectively generate singlet oxygen in these frequency ranges. The analysis of experimental and clinical data suggests that, for being considered as a possible competent photosensitizer, a given compound should be highly soluble in water, in order that injectable solutions or blood substitutes of it can remain stable when stored or exposed to visible light. Additionally, the next requirements must be also satisfied by a proficient photosensitizer:

- To be readily synthesizable, homogeneous, and well characterized,
- To have low toxicity at therapeutic doses subjected under light or darkness conditions,
- To combat high tumor accumulation and facilitate its fast elimination
- To show absorption bands in the wavelength region at which radiation penetration of biological tissues is deeper (*i.e*., far red and near-infrared wavelengths)
- To generate a high quantum yield of singlet oxygen *in vivo*

Currently, tetrapyrrolic macrocycles, especially synthetic porphyrins and phthalocyanines are exhaustively studied as photosensitizers for PDT; however, as it was said above, the next fifth story reinforces the idea that Nature allows for no mistakes. Three thousand years ago, Egyptian and Indian people employed vegetable and plant substances for producing photoreactions in skin that caused repigmentation of depigmented skin lesions, *i.e*., these materials were used as photosensitizers. The photosensitizers employed in these ancient therapies have now been characterized by modern science as members of the *psoralen* family of chemical compounds, which are used today in photodynamic therapy to treat diverse skin illness such as acne, eczema, psoriasis, neurodermitis, vitiligo, cutaneous T-cell lyphoma, *etc*. [109,110].

The idea of delivering a toxin to an organism for inducing a determinate illness, through substances selectively absorbed by this organism (*i.e*., the magic bullet), was pointed out at the end of the 19th century by Ehrlich [111]. As this author described in 1885 in *The Need of the Organism for Oxygen*, several organs of laboratory animals had been colored to different degrees after their deaths by injecting pigments such as alizarin blue and indophenol blue. Ehrlich found that these organs were endowed with high oxygen concentrations, while indophenol was retained and only reduced at intermediate O_2_ concentrations; in turn, alizarin was not altered and both previous pigments were reduced when the O_2_ concentration was low. Ehrlich employed methyl blue, for staining bacteria (methylene blue was previously used by Koch in his research on the tuberculosis pathogen) in 1889, and after realizing that the accumulation of the previous pigment caused the death of bacteria, he conceived the idea of using it therapeutically. Fever subsided indeed and malaria plasmodia disappeared from the blood of two patients treated with methylene blue at the Berlin-Moabit Hospital. There, helped by Hata, Ehrlich discovered in 1909 that arsphenamine (compound 606) effectively combated *Spirillum spirochaetes* bacteria, one of which subspecies that causes syphilis, a terrible and incurable disease of the first last few centuries. The spirochetes disappeared in seven syphilis patients who had few side effects and after extensive clinical testing, the Hoechst Company labeled the healing compound as *Salvarsan* in 1910 [112]. Ehrlich received the Nobel Prize in Medicine for his contributions to immunology in 1908.

The first mention of the potential therapeutic effects of photosensitive dyes, combined with a light source and oxygen, was made in 1903, by Raab, a student of von Tappeiner, who observed the toxic effects of acridine on *Paramecia* cultures, first under a light source [113] and subsequently, after realizing the requirement of oxygen, von Tappeiner defined the event as photodynamic action [114]. Later, Blum [115] applied this definition to the photochemical reactions in which oxygen is consumed. Afterward, von Tappeiner and coworkers developed a treatment for healing skin carcinoma by using a 1% eosin solution and a long term exposure either to sunlight or UV lamp light [116,117]. Four of the patients treated showed total tumor dissapeareance, but suffered a relapse in a period of 12 months.

Here appears the fifth parallel history, when in 1908 Hausmann established that hematoporphyrin, was an active photosensitizer against *Paramecia* and *Erythrocytes* [118]. In 1910, this researcher injected hematoporphyrin to mice then observing that their reactions and effects depended on the type of photosensitizer and light dosing, and postulated that the primary effect was associated with peripheral vessel damage.

In 1912 Meyer-Betz was the first scientist to study the effect of hematoporphyrin on human beings by injecting himself intravenously 0.2 g of this substance, followed by a posterior exposure to sunlight, which caused him an edema and hyperpigmentation that persisted for 2 months [119]. As it was later confirmed, hematoporphyrin produces a violent photosensitization of skin and other tissues.

After observing the red fluorescence of hematoporphyrin accumulated in sarcomas of laboratory rats created by the action of ultraviolet light, Policard in 1924 was the first to suggest the diagnosis capacity of this substance [120].

Nonetheless, in 1948, Figge *et al.* [121] demonstrated that porphyrins exhibit a preferential affinity toward rapidly dividing cells, including malignant, embryonic, and regenerative cells of laboratory animals. Under ultraviolet radiation, the brilliant red fluorescence displayed by the tissue of cancerous tumors produced by injecting hematoporphyrin 12 to 72 hours before the surgery, Figgs and Rasmussen [122] proposed in 1954 that hematoporphyrin should be used as a contrast probe for revealing the size of invisible tumors. Since hematoporphyrin is in reality a mixture of porphyrins and impurities, Schwartz prepared, in 1955, the first hematoporphyrin derivative, HpD, through the treatment of hematoporphyrin with concentrated sulfuric and acetic acids [123,124]. In 1960, Lipson carried out investigations to find an agent for the detection of tumors in patients characterized by HpD [125,126,127,128] and used it to diagnose tumors, even if this substance turned out to be twice as toxic while producing a two-fold photodynamic effect when compared to the original compound. Initially, the investigation was focused on the use of HpD as a fluorescent diagnosis agent, but in 1966 Lipson *et al.* [127] informed of the destruction of malignant tumors in patients with extended and recurrent ulcerated mammary gland cancer, which were exposed to xenon-lamp radiation after HpD was injected to them. In 1972, photodynamic therapy was used for destroying gliomas, implanted in rats after being injected with HpD and exposed to white light [129]. By using HpD again in a similar process, Kelly and Snell observed in 1976, a pronounced destruction of human bladder malignant tumors implanted in mice [130]. As a consequence of this, these authors proposed the possibility of applying a similar treatment for eliminating superficial transitional cell carcinoma from the urinary bladder in humans; this treatment that was performed one year later with a patient suffering of a relapsing superficial anaplastic carcinoma of the urinary bladder.

The photodynamic therapy resurged when Dougherty *et al.* [95,96,97,131,132,133,134,97,131] informed over the total or partial elimination of 111 among 113 cutaneous and subcutaneous malignant tumors. These tumors were treated by means of a tunable dye laser endowed with argon pumping delivered by optical fiber while choosing HpD as photosensitizer. Afterwards a HpD mixture containing 20% of hematoporphyrin and 30% of mono- and didehydrated hematoporphyrins (as for instance hydroxyethylvinyldeutero porphyrin) [125,126] and, as it was found, some of these compounds are inactive or have low photodynamic activity, while the rest contain porphyrins linked through ether bonds to form two to eight pyrrole rings complexes. The preparation known as *Photofrin II, Porfimer Sodium*, or *Dihematoporphyrin Ether* (DHE) [133], which contained at least 80% of active fractions, was approved clinically by the U.S. Food and Drug Administration (FDA) and passed to the third phase of medical probes sponsored by Photomedica, Inc. and, The American Cyanamid Lederle Laboratories (USA), and Quadra Logic Technologies (Canada). In 1993 *Photofrin*, was the first potosensitizer approved to be used in PDT of bladder cancer in Canada, since it provides good results in the treatment of different malignant tumors and it is now the most recognizable photosensitizer in the world. Administered to animals, *Photofrin II* is accumulated by all organs and tissues of the reticuloendothelial system (liver, kidney, and spleen) and also collected by tumor cells at lower concentrations for a longer time, if compared to healthy tissues (12 weeks in rats). Due to the accumulation of these photosensitizer substances in the skin of patients, these remain heliophobic for 4 to 6 weeks for which it is necessary to inhibit sunburn [134].

In 1990 *Photochem*, another photosensitizer derived from haematoporphyrin was developed at the Lomonosov Moscow State Academy of Fine Chemical Technology by Mironov *et al.* [135,136,137,138,139]. This potosensitizer was clinically tested from 1992 to 1996 then showing complete tumor resolution in 92% of the patients. In Russia PDT was applied at the Moscow Medical Center from 1992 to 2001, to treat malignant tumors in 408 patients, with diverse types of cancer, including skin, mammary, oral, esophagus, stomach, urinary bladder, and rectum. The published results showed beneficial effects in 94.4% of the patients, with a 56.2%, and 38.2% of total and partial tumor resolution, respectively. Also, PDT was applied as antimicrobial treatment for drug resistant MRSA and TB infections. Since 1990 China has been acquiring remarkable PDT experience and skill for the resolution of difficult to reach tumours with PDT by using their own photosensitizers, derived from haematoporphyrin [140,141]. The data reported in the literature from 1990 to 2001 showed similar high percentages for total and partial tumour resolution of diverse cancers, as it was previously observed in Russia.

In 1994 a research group headed by Vorozhtsov [139,142,143,144] developed at *NIOPIK*, a Moscow Research and Production Association, the second generation of photosensitizers [142,143,144,145], consisting of water solutions of sodium salts of aluminum di- to tetra-sulfonated phthalocyanine (another member of the tetrapyrrolic macrocycles) named *Photosense * [142,143]. These solutions have strong absorption in the red region of the spectrum with a maximum at 675 nm and a Soret band at 350 nm. Compared to HpD, these species offer some advantages such as a higher photodynamic activity in the red region that is activated by optical radiation that penetrates profoundly in biological tissues thus resulting in adequate treatment of deep tumors. Because of the water solubility shown by phthalocyanine compounds, a single dose ranges from 1 to 2 mg per kg of the patient's weight. A sterile isotonic sodium chloride solution containing a 1:4 proportion of aluminum sulfonated pthalocyanine it can be injected in dark conditions 24–48 hours before tumor irradiation. As what occurs with HpD, patients remain heliophobic for 4 to 6 weeks after treatment. An injectable 0.2% solution of *Photosense*, was tested at several Moscow Research Institutes and employed in PDT of malignant tumors, although the same substance can still be used in the treatment of other non-malignant tumors, ulcers, and festering wounds.

In the last twenty years a growing interest has been developed toward tetrapyrrolic compounds (chlorophyll derivatives, synthetic porphyrins, and phthalocyanines), especially concerning the increase in the selective accumulation of a given photosensitizer in tumors to reach better effects, through PDT. The structure of tetrapyrrolic macrocycles and their inherent physicochemical behaviour makes possible the synthesis of new compounds with useful specific properties applicable in PDT, which have been tested in recent years, with higher tumor cells selectivity and cytotoxicity, but there still exists an especial interest toward photosensitizers that can be rapidly accumulated and eliminated of the tumors, and an especial interest exists for tetrapyrrolic macrocycles acting as photosensitizers, such as porphyrins, chlorin, normal and etiopurpurin, texaphyrin benzoporphyrin, naphthalocyanine, and phthalocyanine. Some day a bank of tumor-targeting photosensitizers will be created (as it has already been made for tumor chemotherapy). Such tumor-targeting photosensitizers will be effective for specific nosological and histological forms of cancer.

In 1942, Snyder [146] suggested the possibility of using water soluble chlorophyll derivatives for medical purposes and non-toxic chlorin mixtures mainly made of chlorin p6 were prepared and administered orally or injected, showing anesthetic, antirheumatoid, antisclerotic, and hypotensive effects. Furthermore, these preparations produced a favourable effect on the biochemical indices of blood, when administrated for 30 days, a decrease of cholesterol in blood, and they were also effective to treat cardiovascular diseases, atherosclerosis, and rheumatoid arthritis. The pheophorbide derivatives were the first chlorin-type derivatives patented to be used in PDT in 1987 [147]. From that year diverse chlorine-type, purpurins and other tetrapyrrole compounds have been patented as potential PDT photosensitizers [148,149,150,151].

The investigations performed from 1994 to 2001 in Russia established the structural and functional features of tetrapyrrole chlorin-type macrocycles (chlorophyll a derivatives) that were needed because of their accumulation in tumors and to increase PDT efficiency. As a result, the second generation of photosensitizers appeared, branded as Photochlorin [148] and Photodithazine [144], which were composed of three cyclic chlorin-type tetrapyrroles, having chlorin e_6_ as the main component (80–90%). Photochlorin and Photodithazine are highly water-soluble compounds, showing good fluorescence which can be activated by light of 654 to 670 nm, which can penetrate about 7 mm in biological tissues and thus inducing high quantum yields of singlet oxygen and producing high phototoxicity. Furthermore, these compounds are stable under darkness and can be stored in darkness at temperatures from 4 to 8 °C, while retaining all their whole properties during 18 months. The chlorin type photosensitizer features better toxic effects, if compared to porphyrin oligomeric and sulfonated phthalocyanines and is eliminated much faster from the body. Photosensitizers such as Photosense and Photohem [138,142] must be applied a long time before irradiation and are retained for more than 3 months; however, chlorin-type photosensitizers only need to be injected 3 hours before irradiation and are eliminated within 2 days. Recently, the Photochlorin commercially named as Radachlorin^®^, a derivative of chlorophyll α, consisting of sodium chlorin e_6_ (90~95%), sodium chlorin p_6_ (5~7%), and a third chlorin, which cannot be disclosed (1~5%), has been showing promising PDT properties for PDT in treatment of cervical cancer [152].

Based on the accumulated experience with the PDT of cancerous tumors, the antimicrobial photodynamic therapy (APDT) [153,154] concentrates on the intercellular interaction between an activated photosensitizer and infectious agents *in vivo*. In this way, the effect of PDT on *Staphylococcus aureus, Streptococcus pyigenes, Clostridium perfingens, Escherichia coli, Micoplasma hominis*, Gram-negative germs and yeast fungi, has been reported by diverse authors [155,156,157,158,159,160,161,162,163]. These authors demonstrated that most Gram-negative and Gram-positive bacteria and fungi can be effectively photoinactivated by water-soluble phthalocyanines or by substituted porphyrins [158,161,162,163]. A century later, the same idea conceived by Ehrlich [111] appears as a possible option for a modern menaces, since it was found that methylene-blue photoinactivates naked viruses such as HIV [164].

In 1990 aluminum sulfonated phthalocyanine was used to destroy *Helicobacter pylori* when it was exposed to a laser radiation of 675 nm, without having ill effects on the mucous membrane [165]. From 1992 to 1998 the inactivation of Helicobacter pylori with PDT and photosensitizers such as methyl blue, toluidine blue, and HpD dyes was studied. The results showed that laser radiation at a dose of 50 J/cm2 produced no damaging effects on the mucous membrane of the stomach, and the bacteria were effectively inactivated at doses of 50 and 200 J/cm2; nevertheless, it was methylene-blue dye which produced the best photoinactivation when irradiated at 50 J/cm2. In 1996 Milson *et al*. explored the possibility of treating the gastric *Helicobacter* through PDT [166,167,168] and investigated in 1998, the photoinactivation of these bacteria *in vitro* by using toluidine-blue, protoporphyrin IX, HpD, and aluminum phthalocyanine, together with different lasers. These authors found that HpD and phthalocyanines attained moderately good bactericide results, even in the absence of laser radiation [167].

At present, scientists are searching for ways of increasing the selectivity and accumulation of photosensitizers in infectious agents, according to the general concept of PDT treatment of tumors. In this way, increased PDT effects over *in vivo Escherichia coli*, were observed when methylene-blue-based was employed in combination with a 1-mA electric current [169] or when a previous laser irradiation was applied. Similarly, the antimicrobial PDT efficiency was increased by employing photosensitizers conjugated with antibodies, such as chlorin e6 linked to poly-L-lysine, which showed a high *in vitro* efficiency against the pathogenic microflora of the oral cavity [170]. As Devanathan *et al.* [171] demonstrated, the use of conjugated bacterial antibodies and isothiocyanate; fluorescent dyes commonly employed as diagnostics agents, produced photoinaction of Escherichia coli and Salmonella bacteria when these were irradiated with ultraviolet radiation (450 to 600 nm). The antimicrobial PDT effects are attributed to the generation of singlet oxygen and peroxide radicals, proceeding from extracellular and intracellular photosensitizers, the action of which brings about a chain of phototoxic reactions. As a consequence of this, new synthetic or natural tetraphyrrolic macrocycle derivatives have been extensively considered as contrast agents or photosensitizers in PDT of tumors since these species manifest significant uptake and selectivity toward cancerous cells if compared to normal and healthy tissues. This last history apparently reveals that, inclusive in tetrapyrrolic macrocycles, man has found again answers and options to combat ancient menaces such as cancer, infections, and viruses.

## 2. Synthetic Porphyrins and Phthalocyanines

**Figure 2:**
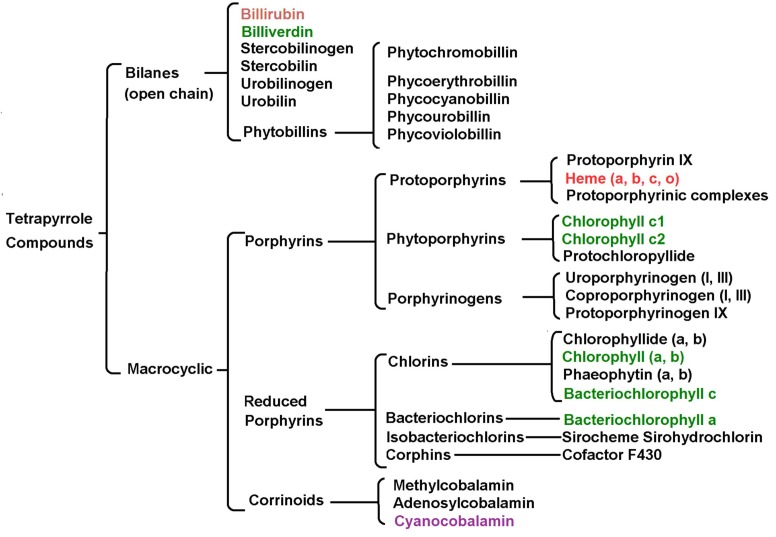
Natural tetrapyrrole compounds can be classified as open chains or *bilanesand* macrocyclic tetrapyrrolic compounds.

As Küster, Piloty, Willstäter or Fischer showed that there exist four pyrroles in the structure of haemin. Similar results were found in the structures of chlorophyll, cytochromes, and cyanocobalamin. At present, it is known that these interrelated structures are members of a larger family of substances derived from pyrrole, the preponderant members of which are the tetrapyrrolic compounds perhaps due to their structural implications. The natural tetrapyrrole compounds can be made of open chains, or bilanes, and cyclic or macrocyclic compounds (Figure 2) [172]. 

Prominent members of bilanes are the heme breakdown products such as billirrubin, and biliverdin; a green tetrapyrrolic bile pigment (derived from the heme catabolism), or the phycobilines found in cyanobacteria. The macrocyclic tetrapyrrole group includes the normal and reduced porphyrins and the corrinoids. As prominent members of these groups are included the heme group (a protoporhyrin), the most abundant chlorophylls a and b, the bacteriochloriophylls (chlorins), and the vitamin B_12_ (a corrin).

**Figure 3:**
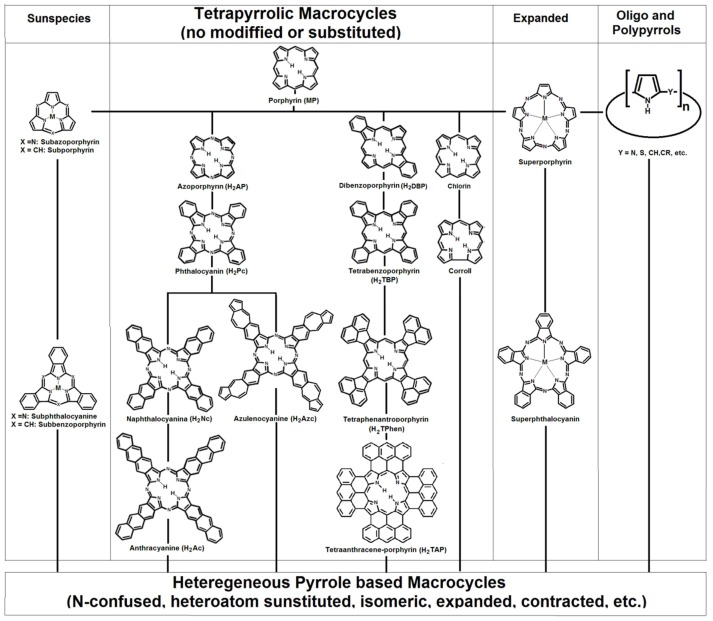
Tetrapyrrolic macrocycles, such as porphyrins, phthalocyanines, naphthalocyanines, chlorines, and corrolls, can be classified as homogeneous pyrrole derivatives. The linking lines drawn only interrelate structurally similar species; for clarity, the nonsymmetric, modified or/and substituted members of this family have been omitted.

With the exception of corrins, in normal or reduced porphyrins the four pyrrolic rings are interconnected through methine groups (Figure 3). The existence of two pyrrolic and two aza nitrogens at the center of the fundamental structure of porphyrins (H_2_P) allow them, to act as a divalent anion or as a tetradentate ligand.

Synthetic compounds such as phthalocyanines (H_2_Pc) [173,174,175,176,177,178,179], tetrabenzoporphyrins (H_2_TBP) [180], tetrazaporphyrins (H_2_AP) [180], and naphthalocyanines (H_2_Nc) [174] display properties similar to that those observed in porphyrins, due to the existence of four central nitrogens in their structures. While, omitting peripheral substituents, natural porphyrins, chlorins, corroles, and related compounds can be included in the enormous family of the tetrapyrrole macrocyclic compounds (Figure 3).

It is interesting to mention that the first phthalocyanines and naphthalocyanines were accidentally synthesized and their interesting properties observed, and with the progress of organic and inorganic synthesis methodologies new macrocycles containing less or more pyrrole units could be obtained, such as the sub*-* [181] and super*-*porphyrins or phthalocyanines [182,183,184]. Due to the possibility of interconnecting pyrrole rings through different groups, expanded tetrapyrrole macrocycles have been synthesized [185,186,187,188,189]. Due to all the physicochemical and technological implications of the natural or synthetic pyrrole derived compounds, new compounds such as synthetic bilanes [190] and biliverdins [191], as well as oligo- or polypyrroles [192] have been synthesized. 

Also, structures in which pyrroles are linked in a different way than in porphyrins, or in which some of them are substituted by other rings, have been synthesized and studied [193,194,195,196,197,198,199]. Derived from the interest in exploring the possibilities of the technological applications of these kinds of compounds, amazing structures have been produced (Figure 3), such as anthracyanines (H_2_Ac) [200,201], azulenocyanines (H_2_Azc) [202], tetraphenanthroporphyrin (H_2_Tphen) [203], tetraphenyltetra-acenaphthoporphyrin (H_2_TPTNP) [107] and, more recently, the extraordinary tetraanthracene-porphyrins (H_2_TAP) [204]. Presently, there now exist a great number of free bases or their respective compounds with D_4h_ symmetry; however, new species with D_2h_ symmetry [205] have been synthesized in relation to dibenzoporphyrin [205]. In 2000, the First International Conference on Porphyrins and Phthalocyanines (ICPP) was organized in Dijon, France and The International Porphyrins and Phthalocyanines Society was founded; this society coordinates the *Journal of Porphyrins and Phthalocyanines*, in which there continuously appear reports on the synthesis and properties of these molecules and related compounds. Due to the importance of these tetrapyrrole macrocyclic species, the structural analogies and their properties as have been carefully analysed [206].

### 2.1. The Porphyrins

**Figure 4:**
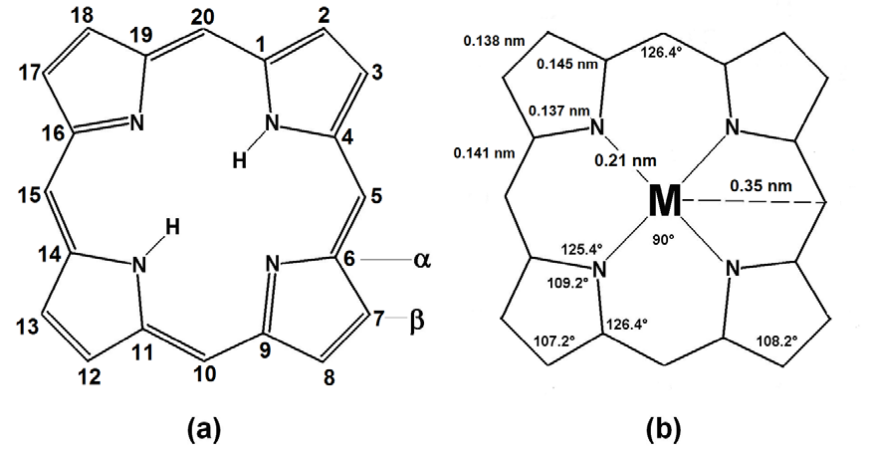
Structures of: (**a**) the tetrapyrrolic macrocycle of porphin and, (**b**) porphyrin, including the principal dimensional parameters and the positions of the possible sustituents.

Formally, porphyrins are aromatic tetrapyrrolic macrocyclic compounds derived from porphin (Figure 4) and while differing from it by the presence of substituents in at the pheriphery of the macrocycle. As it was mentioned above, porphin consists of four pyrrole rings bonded through methine bridges to form a highly conjugated macrocycle with 18 of the 22 π electrons participating in the delocalization, which is in concordance with the Hücklel rule for aromatic compounds. In the free bases of porphyrins the two pyrrolic hydrogens can be substituted by a cation and the other two nitrogens tend to easily coordinate with a metal nucleus to form a stable metalloporphyrin. The metalloporphyrins can be considered as a combination of a cation with the porphyrinate anion P2−, which is a rigid, tetradentated and divalent ligand in which the negative charge is delocalized through the conjugated π electrons system of the macrocycle, which induce extraordinary chemical and thermal stability to the formed complexes.

As it was mentioned above, the porphyrins are strongly colored and stable compounds, but their solutions are unstable in light. The porphyrin macrocycle is stable in sulphuric acid (employed for the elimination of metallic elements in metalloporphyrins), but can be destroyed with perchloric, chromic, and hydrogen iodide as well as with potasium permanganate and other strong oxidants. Because of its closed structure, the central space of the porphyrins can only accommodate ions with an atomic radius smaller than 0.201 nm [207], thus causing that in the porphyrinic complexes with larger ions, the metal must be located out of the molecular plane. Similarly, the size of the cation, or the presence of massive axial ligands, can induce the loss of planarity or the structural deformation of the macrocycle. Furthermore, the peripheral and remaining hydrogens of the pyrroles and those allocated on the methine bridges can be exchanged by different substituents to render an extraordinary family of compounds.

The first unusual porphyrin, a tin porphyrin, was synthesized by Milroy in 1909 [208] and in 1913 Willstäter and Forsen reinserted magnesium in a porphyrin derivative of chlorophyll [24,25,26]. Based on the Fisher investigation, Treibs [209] wrote a magnificent monograph related to the synthesis of diverse cations. Up to 1974, the aim of increasing the number of metalloporphyrins with many main elements of the periodic table was well under way, principally due to the efforts of inorganic chemists who have an intuition about the enormous potential of these compounds. From the middle of the 1960s and to right the end of the 1970s authors such as Tsutsui reported the synthesis of porphyrins with Ti, Cr, Tc and Re [210,211,212]; in turn, Fleischer did the same with Ru, Rh, Ir and Mo [213,214], Treibs with As, Sb, and Bi [209] while and Horrocks was the pioneer of the synthesis of porphyrins with lanthanide elements [215,216,217,218,219]. The interest in these compounds was due to their large magnetic anisotropies and extremely short spin-lattice relaxation times, which made them excellent shift agents in nuclear magnetic resonance. For example, in 1975, Horrocks *et al.* reported the synthesis of thorium or yttrium porphyrins and the incorporation of yttrium into myoglobin [216], which made possible the use the of fluorescence microscopy to investigate the properties of that molecule. 

Buchler was the first to synthesize the porphyrins of Sc, Zr, Hf, Nd, Ta, W, and Os [220], and after that developed the methods to obtain complexes with two or three porphyrins [221,222,223]; Lux reported the synthesis of porphyrinic complexes with actinide elements such as Th, Ac and U [182,183], and Buchler was the first to synthesize the bisoctaethylporphyrins and bistetraphenylporphyrins from practically all the lanthanide elements [222,223]. From the X-ray analysis of these compounds, it was found that the macrocycles are separated around 0.34 nm and rotated 45° one with respect to the another [224,225]. In these bisporphyrinic complexes, one macrocycle is monoanionic and the other one is dianionic with an unpaired electron present in one of them.

The luminescent properties of the ytterbium mono-porphyrins made them very attractive to provide immunity because these molecules showed great Stokes shifts and long life times emission bands in the red and near infrared regions [226,227,228], far of from the typical emission zone of the physiologic endogen fluorophores. These properties made theses species interesting physiological probes, which could also be bonded to diverse analytes to perform radioimmunoassays [229]; the antibacterial effects of these substances have also been studied [230,231].

Synthetic porphyrinic complexes, including practically all the metallic elements of the periodic table have been synthesized [42,43,179] and diverse and applicable properties have been found in them. The extensive delocalized π-electron systems of these centrosymmetric compounds confers upon them, besides chemical and thermal stability, other interesting spectroscopic, optical, and electrical properties that allow a great number of applications in modern technology [232,233,234,235,236]. Great attention has been conferred to the porphyrin free bases since these species are excellent hole-burning photochemical pigments that can be used as high-density storage devices.

As it was mentioned in the first paragraphs of this work, in order to perform their functions adequately, the porphyrinic complexes which are present in chlorophyll, hemoglobin or cytochromes, should be immersed in a proper physicochemical environment while acquiring an adequate spatial disposition in order to perform their transcendental biochemical functions.

The selective oxidation of hydrocarbons, ketones, epoxides or alcohols is still an unresolved industrial question, similar to those processes based on the use of toxic metals such as Cr, Mn, Pb, *etc*. The design of synthetic models of monooxygenase, which is an enzyme with cytochrome P_450_ [63,69,70], by employing synthetic porphyrins fixed on solids supports, constitutes an interesting option that has been explored by diverse authors [237,238,239,240].

With these purposes in mind, many researchers have attempted to create simplified structures that can mimic the catalytic or photoreactive properties of the reactive centers that are present in natural structures. Many models have been designed and optimized by researchers through the years for reproducing the properties of hemoglobin, chlorophyll, or cytochromes**. **In a methodical study Collman and co-workers [240,241,242,243,244,245] have undertaken a systematic research that has lasted for about half a century, implementing experimental strategies for obtaining suitable models of the hemo groups and cytochrome reactive centers through the use of synthetic porphyrins. Very important results have been attained from the study of these synthetic structures.

### 2.2. The Phthalocyanines

The first totally synthetic tetrapyrrolic macrocycle having porphyrin parentage, *i.e*., a tetrabenzotetraazaporphine, commonly named as phthalocyanine, was accidentally obtained in 1907 when Braun and Tcherniac heated *o*-cyanobenzamide at high temperature [173]. Similarly, in 1927 Diesbach and von der Weid, found very stable (both thermally and chemically) blue phthalocyanine complexes with strongly bonded copper, when these researchers were trying to prepare *o*-phthalonitrile from dibromobenzene [174]. In 1928, a similar deep blue and insoluble compound was found in the iron reactors used to synthesize phthalimide by the Scottish Dyes Ltd. Corp. of Grangemouth, Scotland (now known as ICI) [173,178].

During the 1930s, Linstead [246] began the specific study of those compounds and found that they are formed from four isoindole units in a highly conjugated system. Because of the high pigmentation capacity of these compounds, Linstead named them phthalocyanines, from the Greek words *naphtha* (rock oil) and *cyanide* (deep blue).

**Figure 5:**
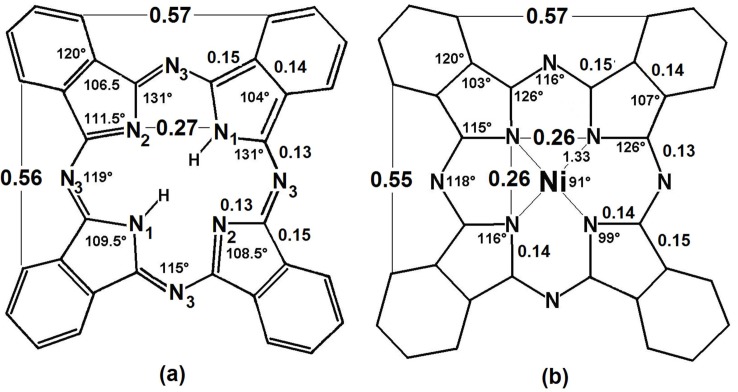
Structure, dimensions and angles: in the free base of phthalocyanine (**a**), and in the nickel phthalocyanine complex(**b**).

From the analyses of the X-ray diffraction patterns of Ni(II), Cu(II) and Pt(II) phthalocyanines, Robertson *et al*. [247] found that in these this kind of complexes, the phthalocyanine ligand molecule is a planar tetra-coordinating ligand with 18 delocalized π electrons. Three types of nitrogen atoms exist in these macrocycles (Figure 5): the pyrrolic nitrogens, (N_1_), the central or aza nitrogens (N_2_) and the bridge aza nitrogens (N_3_), where the central and external nitrogens have very different energies in the phthalocyanine free base of the phthalocyanine, but featuring similar energies in the metallo-phthalocyanine. At that time, the H_2_Pc (C_32_H_18_N_18_) phthalocyanine macrocycle H_2_Pc representation was proposed by Robertson.

As it was observed by Diesbach and von der Weid, the metalized phthalocyanines are thermally and chemically very stable; decomposing above 550 °C, and resisting the attack of Brönsted acids. In 1962, Berezin [248,249] demonstrated that the increased stability of phthalocyanines depends on the ionic radio and charge of the cation, while being demetallized only with sulphuric acid 16 M.

In some documents, such as the review by Lever [175], the synthesis of a large number of metal and metalloid phthalocyanines has been presented, as well as together with a large amount of structural, thermodynamic and spectroscopic data. In a first instance, phthalocyanines were used as highly stable and resistant pigments; the copper phthalocyanine was known as phthalo blue, Windsor blue, copper tetrabenzoporphyrazine, Cu-phthaloblue, PB-15, *etc*. Phthalocyanines were the first organic semiconductors [250], in which the conductivity and optical properties were closely correlated [251,252].

The structures of porphyrins and phthalocyanines are very similar to each other; however, since phthalocyanines are more susceptible to axial union [178,253,254,255,256] and oligomerization, these compounds have been used as models to study: (i) the transport of diverse gases such as O_2_ by hemoglobin (redox reactions), (ii) enzymatic catalysis, (iii) photosynthesis, and (iv) other methabolic processes, such as Weber and Buch [257,258] showed. When an iron (II) tetrasulfonated phthalocyanine carrier is dissolved in aqueous fuming sulphuric acid, O_2_ is fixed to its structure**.** Furthermore, Co(II) tetrasulfophthalocyanine, supported on a graphite electrode and immersed in alkaline medium, can reduce O_2_ when a required voltage is applied [259].

The superconductivity of organic or inorganic polymers, especially those having unidimensional structures, is a scientific field of great interest. In general, the materials used should be highly polarizable electro-attractors or electro-donants with planar structures. As it was previously mentioned, phthalocyanines are planar macrocycles that can form unidimensional compounds. Because of this reason, phthalocyanines are very attractive candidates to be used as donors in superconducting materials, and to in the synthesis of systems labelled as molecular metals [260,261,262,263,264,265,266,267].

Because of their symmetric structure and the presence of four central nitrogens, it is possible to obtain complexes with more than one pthalocyanine macrocycle. The first complex having two phthalocyanines bonded to tin (IV) ion was synthesized by Barret in collaboration with Linstead in 1936 [268], but it was not until 1965, when Kirin y and Moskalev [269,270,271,272,273,274,275] reported obtaining similar complexes of praseodymium, neodymium, erbium, and lutetium. The synthesis was made through annealing of a 1:8 mixture of lanthanide acetate, Ln(Ac)3•nH_2_O, and dicyanobenzene (DCB), from 280 °C to 290 °C. In 1967, the synthesis of complexes formed by two lanthanide ions and three phthalocyanine macrocycles, Pc3Nd2 was reported by the same scientists [270], and in 1970 [271] a method was proposed to obtain only bisphthalocyanine HLn(Pc)_2_ complexes, through the use of a mixture of 1:10 of Ln(Ac)3·nH_2_O and DCB heated at 300 °C. As the same authors stated, bis- or tri-phthalocyanine complexes can be obtained when the tri- or tetra-valent central ion has an ionic radius larger than 0.183 nm; a value that is very similar to the size of the central window of the phthalocyanine macrocycle. With the above expressed idea, Kirin and Moskalev [272,275] and Lux and Dempf [182,183], initiated the research for obtaining actinide bisphthalocyanines.

Surprisingly, tin, with a covalent radius of 0.14 nm, can form a bipthalocyanine complex. The X-ray patterns showed that in lanthanide bisphthalocyanine complexes, the two macrocycles were bonded to the central lanthanide ion [276,277] and separated from it by around 0.3 nm and rotated 45° with respect to one other. In this sandwich structure, one of the macrocycles shows a folding of around 13° with respect to the horizontal molecular plane, which was initially attributed to a hindering effect. Nonetheless, as it was later demonstrated, through magnetic susceptibility, this deformation is caused by the existence of an unpaired electron, probably localized in the deformed macrocycle [276,277]. In 1972, Kirin and Moskalev observed an appreciable color change in luthetium bisphthalocyanine deposited on an electrode when a potential of 0.0–1.0 volts was applied [273]. By 1975, these same authors claimed that these compounds [276,277,278,279] are very sensible to pH, and the UV-Visible spectra changed markedly from an acid to a basic environment. This fact was attributed to the proton loss in one of the macrocycles. Besides, this observation suggested the possibility of using sulfonated lanthanide bispththalocyanines as acid-base indicators [273,274,277].

In 1977, Nicholson *et al.* [280,281,282,283] demonstrated the interconversion of among neutral, mono or dicationic forms of HLu(Pc)_2_; every one of these having a different color, through the successive elimination of electrons by the application of an electrical potential of about 1.2 volts. This interesting phenomenon, called electrochromism, results very useful in graphic techniques and electronic displays [280,281,282,283,284,285]. Additionally, neodymium or lutetium bisphthalocyanines have also been largely studied by the same authors [283], since, as it was mentioned above, the phthalocyanines are usually semiconductors [250,251].

Due to all of the above interesting properties and structural coincidences with natural tetrapyrrolic macrocycles, synthetic species such as phthalocyanines, porphyrins, and related species have been tested and investigated to be used as probes [216,218] immunoassays [286,287,288,289,290] and also as possible photosensitizers in PDT treatment of diverse cancers [99,100,101,291,292,293,294,295,296,297,298,299,300,301,302,303,304,305,306].

### 2.3. Porphyrins and Phthalocyanines in Catalysis and Sensoring

Because of their great thermal and chemical stability, the phthalocyanines can be used in different catalytic reactions, as oxidants in the liquid phase [307,308,309,310] or gas phase [311,312,313,314]. The catalytic activity of the metallic phthalocyanines in oxidation processes is due to their remarkable capacity of axial coordination of O_2_ [57,307,313,314,315,316,317,318]. The poisoning of catalysts with mercaptans is caused by the stronger coordination of these substances over the metallic elements of the substrate [319]. Most times, in macrocycle based catalysts, this phenomenon almost does not ocurr since the macromolecule inhibits the interactions of the central metallic cation with mercaptan. As an example, it is possible to oxidize alcohols through the use of copper phthalocyanines [320], or to reduce oxygen to water with phthalocyanines or porphyrins of Fe(II ó III), Co(II), and Cu(II) [321,322,323], or with polyphthalocyanines of Fe [324].

The phthalocyanines have been used since long ago as oxidation catalysts [325] and, in the 1960s Hara and Katsu [326,327,328,329] employed phthalocyanines of Fe(II) to catalyze the oxidation of tri-tert-butylphenols; finding that phthalocyanines of Co(II), Mn(II), Ni(II), and Cu(II), were inactive in the autoxidation of phenols. Kothari *et al.* [330] found that in the presence of dimethylformamide (DMF), Co(II) phthalocyanine catalyzes the oxidation of dialkylphenols or dialkylbenzophenones. The catalysts with polyphthalocyanines of Cu and Fe have been used to oxidize cumene and acrolein [326,327], as well as aromatic compounds and cycloalkenes; in turn, cobalt phthalocyanine can be used for the sweetening of gasoline [331]. Surprisingly, the free polyphthalocyanine is capable of catalyzing the oxidative dehydrogenation of alcohols, aldehydes, and ketones [332]. Of transcendental energetic implications is the possible use of some porphyrin or phthalocyanin complexes to catalyze the water reduction to H_2_ and O_2_ [333], the epoxidation of cycloalkenes [244] or the sweetening of gasoline [331].

To recreate the optimal physicochemical conditions under which porphyrins, phthalocyanines, or similar complexes catalyze some processes, it is often required to first trap them inside the adequate solid supports to improve their physical and chemical strength. This situation makes possible the recuperation of the materials with their active centers as part of the structure and not only as mere inclusions or trapped species. This entrapping can be made through direct synthesis [334,335,336,337,338,339], by anionic exchange [340], by reconstruction [341], or by impregnation [336]. In this form, Kannan *et al.* [338] or Carrado *et al.* [339] have intercalated copper or cobalt tetrasulfophthalocyanines, CuTSPc or CoTSPc, inside hydrotalcites of Mg-Al. Through direct synthesis, it is possible to trap CoTSPc inside hydrotalcites prepared from a mixture of Mg and Al nitrates heated at 60 °C and dried at 70 °C [331]. The obtained materials show adequate mechanical properties and allow the elimination of mercaptans without a basic medium. Similarly, for effective catalysis applications, Fe(II) or Co (II) complexes of tetraphenylporphyrins, azoporphyrins, or phthalocyanins require firstly to be trapped in carbon in order to be used as catalysts for the reduction of O_2_ in fuel cells [342,343,344] or the oxidation of thiols [345,346].

As mentioned above, the existence of metallic elements at the center of tetrapyrrolic macrocycles confers them extraordinary properties, one of the most interesting being their capacity to axially coordinate different chemical species, such as O_2_ or CO2, as happens with the heme group of hemoglobin and cytochromes. Similarly, the synthetic porphyrins and phthalocyanines display excellent coordination capacities [42,43,178,179], which commonly produce an optical response**.** As Ferraudi or Savitsky reported** [254,347,348]**, the axial ligand in aluminum tetrasulfophthalocyanine, depends on the pH of the medium, which could be detected by fluorescence spectroscopy.

Through the last century it was observed that the tetrapyrrolic macrocycles responded to the physicochemical environment or the presence of different chemical or biochemical species, via a change in color or a spectroscopic signal response. For this reason, the use of macrocyclic compounds as optical and colorimetric sensors has been explored for many years, but though with more enthusiasm in the last decades of the last century. In this way, Lee and Okura [349], developed in 1997 a photochemical optical O_2_ sensor with a fast response time of 5 s, by using platinum octaethylporphyrin, PtOEP, incorporated into a silica substrate, obtained by the sol-gel method. The detection is possible since the phosphorescence of the macrocyclic compound diminishes in the presence of O2. In 2000, Rakow and Suslick [350] developed an optical sensor for volatile substances such as amines and carboxylic acids; these devices were based on the color changes of metaloporphyrins interacting with these substances. As Feng *et al.* [351] recently reported, diverse compounds such as porphyrins respond to the presence of toxic substances with a detectable color change.

Furthermore, Dunbar *et al.* [352], investigated the possible use of seven free bases of tetrakis[3,4-bis-(2-ethylhexiloxy)phenyl]porhyrin (EHO) and its complexes with Mg, Sn, Ag, Au, Co, and Mn, as colorimetric sensors in the gas phase of volatile organic compounds such as acetic acid compounds, butanone, acetylacetonate, hexanethiol, hexilamine, hexane, octanol, octanal, octilamine, triethylamine, and trimethylphosphate. In the year 2000, Worsfold *et al.* [353] performed studies to develop thin films of tetrasubstituted porphyrins trapped in sol-gel networks to detect low quantities of NO2 (4.4 ppm). In a similar way, the properties of porphyrins incorporated into mesoporous solids have been analyzed, as shown by the research made by Balaji *et al.* [354], who developed an optical mercury sensor. The luminescent properties of two porphyrinic complexes of Pt(II) trapped in mesoporous silica as possible sensors of oxygen were studied by Zhang *et al.* in 2005 [355].

In order to employ in a better way the interesting properties of porphyrins and phthalocyanines mentioned above, it is occasionally necessary to trap or fix them in appropriate solid supports. Unfortunately, due to their physicochemical nature, porphyrins, chlorines, corrols or other natural tetrapyrrolic compounds cannot be trapped inside solid porous networks by means of thermal diffusion since traditional methods of this sort render heterogeneous and lowly concentrated materials [334,335,336,337,338,339,340,341]. The sol-gel method emerges as an interesting and versatile option in order to encapsulate sensitive chemical species such as tetrapyrrolic species, or porphyrins inside inorganic oxide networks.

## 3. The Sol-Gel Process and the Trapping of Chemical Species within Xerogels

The sol-gel process was has been labelled as chimie douce [356,357,358] or soft chemistry because procedure it takes place at relatively low temperatures and under no aggressive chemical conditions [334,335,336,337,338,339,340,341]. Through the sol-gel process the inorganic pore networks can be synthesized from: the assemblage of colloidal particles (Method 1) or from the hydrolysis and polycondensation of the appropriate precursors (Methods 2 and 3), such as inorganic salts, organometallic compounds, or metal alkoxides Mn+(OR)n. When water reacts with the Mn+(OR)n species causes a partial or total substitution of the alkoxide groups (OR) by hydroxyl species (–OH); this takes place, through hydrolysis reactions (1), which form metaloxane bridges (M-O-M) through polycondensation reactions among the partially hydrolyzed species (2). As a consequence of the polycondensation, the initial homogeneous mixture renders micrometric particle dispersion called a sol, which turns into a gel as the network grows in size and complexity [356,357,358,359,360]. The gel consists of a tridimensional solid network entrapping a liquid mixture inside their cavities. In the case of silica materials, tetraethoxy- or tetramethoxysilane, TEOS or TMOS, respectively, are the most common precursors that have been employed. Compared with the silicon alkoxides, which can be used with relative facility, the alkoxides of transition metals need to be manipulated with precaution because these react vigorously and exothermicaly with water [356]:

Hydrolysis:

Mn+(OR)n + n.H_2_O → HO- Mn+(OH)x(OR)n-x + xHOR (1)

Condensation:

(a) Aqueous: 2.(HO)x Mn+(OR)n-x → (HO)x-1(OR)n-xM-O-M(OR)n-x(OH)x-1 + H_2_O (2)

(b) Alcoholic:2.(HO)x Mn+(OR)n-x → (HO)x-1(OR)n-xM-O-M(OR)n-x-1(OH)x + HOR (3)

The gel continues increasing its complexity and strength because the polycondensation continues until the mixture gels, due to the formation of more M-O-M unions. For the same reason, at this stage some pores reduce their sizes (shrinkage) and the excess of the liquid mixture is expelled from the pores to the outer surface where it is finally evaporated (syneresis). When the remnant liquid is eliminated from the gel under hypercritical conditions an aerogel is formed, but is named a xerogel or gel, if the liquid is removed under ordinary conditions. The gel is maintained immersed in the same liquid mixture the most possible time or is processed by a suitable thermal treatment to increasing the mechanical resistance of the solid, while additives could still be used employed to reinforce the structure. After some aging, the gel can be densified by annealing it at relatively higher temperatures to produce powders, ceramics, or glasses, thin films, *etc*. [359,360]. However, the preparation of stable, strongly built, and optically adequate monolithic xerogels generated by the sol-gel method is more complicated; and in 1988–1989 methodologies were implemented to synthesize devices based on monolithic silica gels, including the so-called totally dense silica (Type V) or the optically transparent porous silica (Type VI), with properties comparable to those observed for optical quality quartz (Type III and Type IV silicas) [361,362,363].

As it was previously demonstrated by other authors, the presence of H_3_O^+^ ions in solution increases the hydrolysis and condensation rates [364], whereas the presence of OH^−^ ions only affects the condensation rate [365] and the mixtures prepared with acid catalysis are more viscous than those created with basic catalysis [366,367]. The laser light dispersion experiments demonstrated that linear products can be obtained under acid conditions, while ramified ones are attained under basic conditions [368,369]. Furthermore, the hydrolysis and condensation sol-gel rates, and consequently the textural properties of the final gels, depend on the acid or basic catalyst employed, the pH of the initial gelling mixture, and of other factors, principally the water/alkoxide ratio (**h* = η_H_2_O_/η_alkoxy_*), the nature of the precursors, the presence of additives, solvents, the temperature, in a lesser degree, the pressure [361,363,370,371,372]. As a consequence of all this, the range of the *h* ratio as well as of the pH used to prepare the gels substrates are both extremely wide. The time in which the mixture turns into a gel or the gelling time (tg), decreases when the amount of water increases, and as Yamane *et al.* [373] demonstrated, the tg *vs*. pH graph have adopts a Gaussian shape, which signifies that gellification occurs instantaneously under strongly acid or basic conditions. However, two solutions at the same pH could gellify at different times due to the identity of the counter ions of the acids used, which, like the nature of the alkoxides or solvent employed, determines the kinetics of the process [374,375].

The soft conditions usually employed in the sol-gel process allow the physical trapping of diverse inorganic, organic [375,376,377,378,379,380,381,382], biochemical [383,384,385,386,387] and, inclusive living species [388,389,390,391] inside the pores of an inorganic network. Furthermore, the sol-gel method allows the preparation of novel materials such as nanocomposites, powders, ceramics, thin films, monoliths, and translucent substrates endowed with very small pores and without hydroxyl groups attached to the pore surface. These last materials are appropriate for optical applications or to prepare solids with tailored pore size distributions that permit an efficient diffusion of reagents as it is needed required in for the case of catalytic applications.

In the xerogels in which an Eu^3+^ ion was used as the first probe species to monitor the sol-gel process [377], the fluorescence was of a low intensity and short lived, possibly due to the interactions of the lanthanide ion with the -CH or -OH groups attached to the network [376,377,378,379,380]; however, when the system is thermally treated, the lanthanide ion displays its rich spectroscopic properties influenced by the gel structure. By using the changes of the fluorescence intensity of the neutral or protonated forms of the 8-hydroxy-1,3,6-tetrasulfopyrene (pyrene) [378], used as probe to follow the polarity changes induced by the hydrolysis-polycondensation reactions in a gelling mixture, it was found that, at low pH values, the hydrolysis is faster and the polycondensation slower and enduring until the end of the gelling action time.

Pouxviel *et al.* [381] used molar ratio mixtures *h* = 10:1, 20:1 or 30:1 of precursor:H_2_O for synthesizing aluminosilicates with tetrasulfopyrene as a luminescent probe for studying the processes of polycondensation, gelling, and drying of gels. Through this study, the authors demonstrated the existence of interconnected pore channels inside the structure of the xerogel obtained by the sol-gel method.

However, an adequate physical trapping of species inside xerogels has been successfully accomplished by using appropriate gelling mixtures with assorted water/alkoxide ratios (*h = η_H_2_O_/η_alcóxido_*). For instance, Campostrini *et al.* [380] employed a molar ratio *h *= 4:1 of H_2_O:Si(OR)_4_ to prepare translucent gels for studying the thermal diffusion of Eu^3+^ ions inside the resultant network. Levy *et al.* [379] chose a mixture with a molar ratio of H_2_O:TMOS *h* = 20 and spiropyrene for preparing photochromic materials for possible information storage applications. In their research, Canva *et al.* [382] used the molar ratio *h =* 10:1 of TMOS:H_2_O in a mixture with methanol, HNO_3_ and formamide to prepare xerogels containing sulforhodamine 640 for laser applications.

As Dickey found in 1949, those xerogels in which methyl orange was trapped and eliminated, can preferably reabsorb methyl orange and none of those analogues containing ethyl, *n-*propyl or *n-*butyl groups [392]. Cytochrome c is a protein molecule with a molecular weight mass of about 12,400 Da and a heme group present in its structure, and displays a charged surface and/or endowed with various points at which diverse molecules can interact though hydrogen bonds. As Dunn and Zink demonstrated in 1996 [383], the UV-Vis spectra of cytochrome *c* in solution and trapped inside an aged silica xerogel results in very similar interesting materials. The Soret band observed at 406 nm in the solution was also observed at 404 nm in the aged gel and at 395 nm when the gel was dried at room temperature, but the signal returned to 404 nm when the dried gel was submerged in an acetate buffer (p*h* = 4.5). The reversibility of the shift only can be due to the solvent lost during the drying stage and not to a large contraction of the gel, because of the size of the trapped molecule. Although the xerogel shows a varied pore size distribution and the size and shape of the empty pores could represent about 70% of the total volume, those pores containing cytochrome c were different since the protein affects their formation and final structures. The cytochrome *c* remains trapped in pores of the adequate dimensions whose network was formed around the protein proceeding from silicate fragments attracted through hydrogen bonds, which apparently are sufficient to modulate the size and shape of the cavity. The above results concerning the formation of solids with model pores preceded the preparation of nanoporous silica. The supposition that the interactions between the growing cavities and the adequate dopant molecules could make possible the tuning of shape and size of the cavities of forming part of the networks obtained by the sol-gel method was proven to be true [383,384]. The so called porosity engineering [393] is now a very active research field with the pretention of making possible the synthesis of porous materials with well defined (size and shape) cavities for applications in molecular recognition, sensors, catalysis, and selective adsorption.

As mentioned above, porphyrins or phthalocyanines have already been trapped in diverse solid networks for catalytic applications [183,320,321,322,323,324,325,326,327,328,329,330,331,332,333,334,335,336,337,338,339,340,341,342,343,344,345,346,347], and some researchers have applied the sol-gel process for inserting macrocycles inside a variety of pore networks. In this form, Fuqua *et al.* [394,395,396,397] trapped copper tetrasulfophthalocyanine, CuTSPc, inside translucent aluminosilicate xerogels to study the effect of the external environment in the control of the aggregation of the macrocyclic species. With the same species, Litran *et al.* [398] studied the aggregation effects on the non-linear absorption of CuTSPc trapped inside silica xerogels. In turn, Inoue *et al.* [234] studied the optical properties of tetra-sulfo, -carboxy or -pyridinium tetraphenylporphyrins trapped inside thin films of doped silica doped. Similarly, Kamitani *et al.* [399], trapped the free base of the tetra-*p*-sulfo-phenylporphyrin, H_2_T(p-SO3H)PP, through a two-step method, inside the pores of monolithic silica gels. Finally, Wang *et al.* [400] studied the non-lineal optical properties of the copper tetrakis-para-sulfophenylporphyrin, CuT(p-SO3H)PP, trapped in monolithic aluminumsilicate gels. Although diverse investigations were made in the past, it was until the end of the last century, and specifically during the last decade that a serious development of methodologies to covalently bond tetrapyrrole macrocycles to inorganic networks through the sol-gel process was undertaken.The Brazilian group of Serra *et al*. [401,402,403,404,405] was perhaps the most persistent research group in this respect.

In the last twenty years the combined use of sol-gel methods and templating and directing structuration agents has allowed the development of sieve science and induced the discovery of a new family of ordered mesoporous molecular networks, such as the M41S family synthesized by scientists of the Mobil Oil Corporation [406,407]. The pore sizes of these materials ranged from 1.6 to over 10 nm. Furthermore, these mesoporous networks result on create thermally stable inorganic analogs of organic liquid crystalline phases and are induced to be adapted to the chemistry of liquid crystals and to the synthesis of mesoporous materials, in order to tailor the structure and porosity of the sol-gel networks. Depending on the structure of the templating molecule used different phases can be synthesized. In this way, it possible to synthesize the MCM-41 (Mobil Corporation Material 41) mesoporous sieve, which possesses a regular hexagonal array of uniform cylindrical pore sizes from 1.5 to 10 nm [408]. By a similar procedure, but using a different tailoring surfactant species, a cubic array named MCM-48 or a lamellar phase named MCM-50, can be obtained. The use of amphiphilic triblock copolymers as pore templates induces the organization of the silica network, thus resulting in the obtaining of well ordered mesoporous networks, labelled as SBA-n materials (Santa Barbara Material), such as SBA-15 [409], SBA-16 [410] or SBA-1 [411]. The SBA-15 constitutes, with the MCM-41, one of the most important hexagonal mesoporous structures. Some other important members of the M41S mesoporous silica family are MCM-41, MCM-48, MCM-50 and SBA-n sieves, and every one of the above materials shows specific structural characteristics with a well defined pore size range [412]. Through the use of surfactants and organic functionalized silica precursors it was possible to synthesize the periodic mesoporous organo-silicas (PMOs), which can be prepared from micelles surrounded by alkoxides such as bis-(triethoxysilyl)ethane [413,414,415,416], thus resulting in an hexagonal array with the structural characteristics of silica, but the chemical functionality of polymers.

Due to the well defined characteristics of model mesoporous materials, these substrates have potential applications as catalysts, adsorbents, trapping agents, in chromatography, optics, sensors and other interesting applications. In particular, several authors have investigated the possibility of synthesizing hybrid materials for catalysis based on porphyrin and phthalocyanine species trapped or bonded to the metal oxide inorganic mesoporous networks [394,395,396,397,398,399,400,401,402,403,404,405,417,418,419,420,421].

As it was mentioned above in heterogeneous catalysis, metal oxides or mixed oxides employed as catalysts or supports of active species, result crucial for performing a good process and the sol gel process represents a unique technique for the preparation of materials with outstanding characteristics. During the hydrolysis and condensation reactions of the sol gel process, the presence of water results crucial; nevertheless, during the last decade, it has been possible to develop the non-hydrolytic sol gel procedure (NHSG), in which polycondensation occurs in nonaqueous media [422]. The NHSG has been established as a powerful alternative for the design of metal oxides or mixed oxide catalytic materials, with assorted composition, structure, texture, and morphology. This method allows the preparation of hybrid materials such as mesoporous xerogels and single-site catalysts. Additionally, the sol-gel process can be performed under supercritical condition, through the so-called Chemistry in Supercritical Fluids [423]. In this way, metal oxides of different morphologies, such as nanospheres, nanofibers, membranes, monoliths and crystalline nanoparticles can be obtained.

During the last two decades, the development of “biofriendly” sol-gel methods [424], has made possible the immobilization of enzymes, antibodies, functional nucleic acids, and DNA enzymes, as well as the preparation of delicate species such as luciferease, kinases and, even membrane-bound receptors, while allowing the materials produced this way to be used as biosensors. As Livage *et al.* [356,357,358] pointed out, this *chemie douce* procedure is very versatile for the production of biosensors through dip-casting, contact printing, and noncontact inkjet printing. Through these methods, it could be possible to produce optical biosensors, optical fiber sensors, multianalyte sensors, and paper test strips.

Furthermore, nanomaterials such as mesoporous silica nanoparticles, MSNP, which are endowed with large pore volumes and specific surface areas, can be functionalized with diverse chemical species for increasing their versatility toward the controlled delivery of different pharmaceutical compounds [425]. From the early 1990s, some other materials have been synthesized from concentric nanoparticle multilayers to improve the properties of the final substrate, e.g., semiconductivity. As a consequence, these types of nanoparticles (composed by two or more materials) were named core/shell nanoparticles. Semiconducting fluorescent nanoparticles are generally toxic for humans, but the synthesis of core/shell nanoparticles could still improve their luminescent properties as well as their biocompatibility. Thus, the synthesis of particles with useful dual properties, for instance magnetic and luminescent, is a very attractive possibility [426]. The application of the sol-gel process for the synthesis of new hybrid materials, and production of catalyst, biosensors or hybrid materials for drug delivery are modern emerging interdisciplinary areas. The combination of these technologies and the use of tetrapyrrole macrocycles could indeed contribute to the production of new and novel hybrid systems.

## 4. Tetrapyrrole Macrocycles and Cavity Pore Engineering Through the Sol-Gel Method

As a result of all the above mentioned facts, we have performed a systematic study concerning the problem of the physical entrapment or the chemical bonding (in stable form) of tetrapyrrole macrocyclic species, such as porphyrins, pththalocyanines, naphthalocyanines, and related species inside inorganic networks through the sol-gel method. Our principal interest is in the synthesis of solid systems in which these macrocycles conserve as much as possible the properties they display in solution. During the last decade, we have provided convincing evidence concerning the feasibility of properly tuning the size, shape and internal physicochemical environment of the pores forming a growing network through the interactions between matrix precursors and tetrapyrrole macrocycles. The experience of the last ten years induces us to believe that is possible tune the size, shape and the internal physicochemical environment inside the pores of a growing network through the interactions between matrix precursors and tetrapyrrole macrocycles. Our research group has been developing appropriate methodologies to physically trap or covalently bind tetrapyrrole macrocyclic species, such as porphyrins, phthalocyanines, and naphthalocyanines inside the pores of inorganic oxide networks synthesized by the sol-gel method.

### 4.1. Aluminum μ-Hydroxytetrasulfo Phthalocyanine,(OH)AlTSPc as Molecular Probe

**Figure 6:**
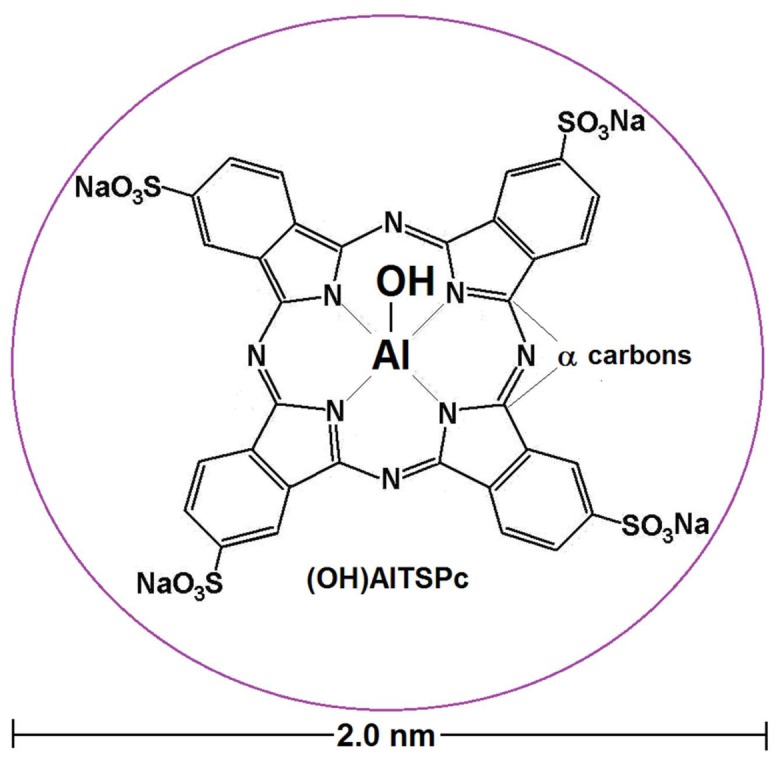
Structure of the μ-hydroxy-tetrasulfophthalocyanine of aluminum.

In the first stage of our investigation, the aluminum μ-hydroxytetrasulfophthalo-cyanine(OH)AlTSPc (Figure 6), one of the species used by Russian researchers as the first compound of the second generation of phothosensitizers, and a species endowed with remarkable thermal and chemical stabilities, was selected as a probe molecule to follow the gelling process and to the determine the adequate proportion of precursors, which that could render translucent monolithic xerogels. The (OH)AlTSPc molecule exists as monomer in aqueous solutions at high concentrations. The UV-Vis spectrum of the (OH)AlTSPc in solution shows two principal bands: a Soret or “B” band at around 347 nm, which is assigned to the a2u(π) →eg(π*) transition, and a more intense QII band, at around 679 nm, due to the a1u(π) →eg(π*) transition [178,427,428]. Due to the presence of a hydroxyl group axially bonded to the central Al atom, the (OH)AlTSPc species usually depicts shows no sign of aggregation, contrastingly, other similar species such as CuTSPc, NiTSPc or CoTSPc strongly tend to dimerize. The respective UV-Vis spectra of MTSPc aggregates show a Soret band at around 350 nm and a Q band at about 630 nm [178,427,428]. The last difference makes possible to distinguish between MTSPc aggregates and monomers. Additionally, (OH)AlTSPc solutions display an intense red fluorescence with a principal band located at 697 nm (λ_exc_ = 350 nm). The quantum efficiency of the fluorescent signal corresponding to the monomeric (OH)AlTSPc species is 0.6 and can even be detected at extremely low concentrations. These last (OH)AlTSPc properties remain relatively constant over a wide pH range (*i.e*., from 4 to 13) and the shifting in wavelength of the Soret and Q bands of (OH)AlTSPc aqueous solutions are indicative of the change suffered by the axial ligand [347]. For this reason (OH)AlTSPc species was chosen as a reference UV-Vis tracer for finding the conditions at which an efficient entrapping of macrocycles inside sol-gel pore networks can be performed [429,430]. 

In this way, it was found that the following molar ratio: 19.6:1:10^−3^:1.1 × 10^−3^ of H_2_O:TEOS:HCl:(OH)AlTSPc is required for effectively trapping the (OH)AlTSPc inside pores in a stable and monomeric form. In these systems, the average pore size was 2.68 nm and the specific surface was 586 m^2^/g [429,430]. By using this methodology and employing adequate inhibiting aggregation agents, such as py and DMF, it was possible to trap in disaggregated form tetrasulfophthalocyanines of transition elements, MTSPc (where M = Fe, Co, Ni, and Cu) inside the pores of translucent and monolithic silica xerogels, which sizes were about one tenth of the initial mixture volume (V_f_). The tetrasufophthalocyanine macrocycle has a size of about 2.0 nm (Figure 6), and the BJH average pore diameters (Φ), determined from the N_2_ adsorption experiments, in the samples in which MTSPc species were trapped, ranged from 2.7 to 3.5 nm [430].

Interesting results were obtained when DMF was used as an aggregation controlling agent in monolithic gels doped with CuTSPc or CoTSPc, which gradually lose their original color, a bleaching phenomenon associated with the degradation of the phthalocyanines. The phenomenon is only seen in systems including complexes of divalent cations, M(II)TSPc which do not possess any axial ligands, by comparison to the M(III) complexes of (OH)FeTSPc and (OH)AlTSPc. Monoliths with (OH)M(III)TSPc species prepared in the presence of DMF or pyridine can entrap in monomeric or in the μ-*oxo* dimer forms, respectively [431].

**Figure 7:**
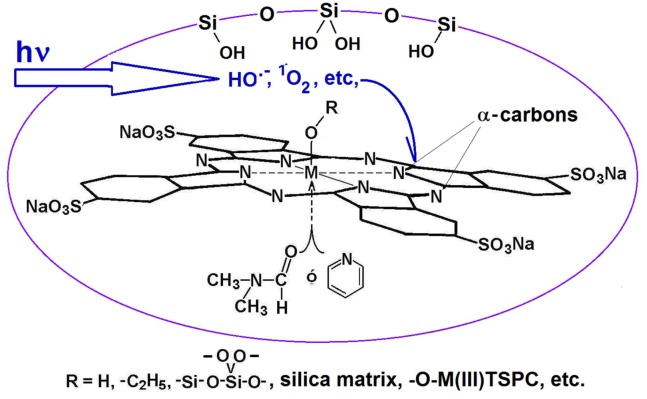
Hypothetical degradation mechanism of MTSPc species trapped inside the pores of silica through the photogeneration of radical species.

The oxidative decomposition of phthalocyanines is a well known phenomenon [178,246,325] and Pedersen [432] showed that the a-carbons (Figure 6) are the most reactive sites in these processes. As Mathur *et al.* reported [433] the charge density is a minimum at sites a1 and a2 of the phthalocyanine molecule. When the a-carbons are attacked by nucleophilic species, the phthalocyanine molecule can then be folded, thus destroying the extensive resonance of the planar macrocyclic molecule without breaking it. However, it is known that cobalt and iron phthalocyanines undergo rapid destruction in aqueous alkaline solution [431,434] and that some other macrocyclic compounds such as the porphyrins and the benzoporphyrins can photobleach other species by photo-oxidative processes involving singlet oxygen [435]. In the gels, this destruction can be attributed to radical species photogenerated from the residual oxygen trapped in the gelling mixture, either by ethoxy ions coming from tetraethoxysilane (TEOS) or by hydroxyl species of the silanol groups attached to the interior walls of the pores, assisted by the DMF molecules. The present theories about the mechanisms involved in the photodynamic therapy (PDT) concern themselves primarily with oxygen-centered species, mainly singlet oxygen and the hydroxyl radical, OH^•−^ [303]. Recently, Ishii *et al.* [436] demonstrated that similar systems based on phthalocyanines supported in silica, are capable of producing singlet oxygen by the photodesorption of molecular oxygen from the network. This event induces us to suppose that, in our systems, the proximity of the MTSPc species with to the pore walls could promote the production of radical species, which turn cause the MTSPc degradation (Figure 7).

Thus, the study of the degradation mechanism of phthalocyanines and porphyrins trapped or bonded to the pore walls of the gel [431] can be used as a model in the study of the potentiality of these and other molecules in phototoxic or photodynamic therapies [303,304,305,306]. Inclusively, the possibility exists of synthesizing photoreactive systems to produce singlet oxygen through the trapping or bonding of tetrapyrrolic macrocycles inside the cavities of inorganic or hybrid networks.

**Figure 8:**
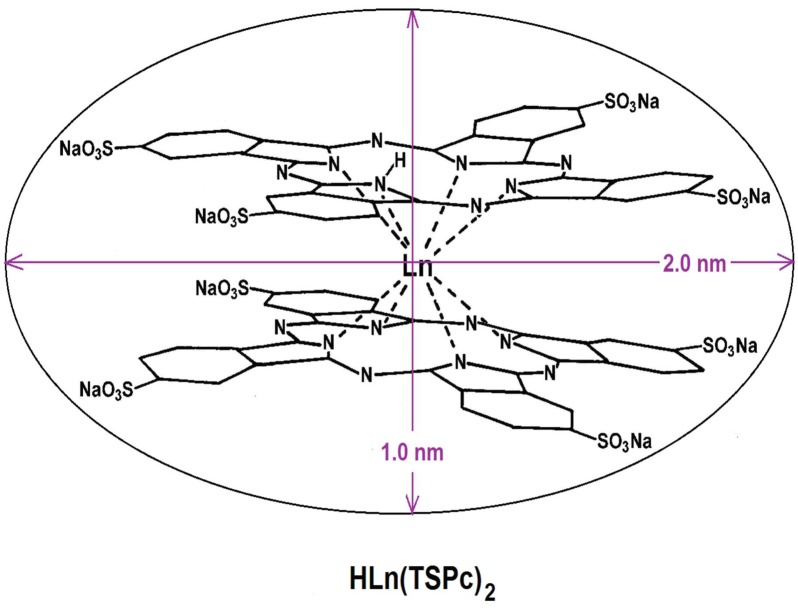
Structure and approximate sizes of the cavity in which a lanthanide bis-tetrasulfophthalocyanine, HLn(TSPc)_2_ is trapped.

As we observed, lanthanide bis-tetrasulfophthalocyanines, HLn(TSPc)_2_, solutions display fluorescence, featuring a maximum wavelength depending on the identity of the lanthanide species. As mentioned above, in these HLn(TSPc)_2_ complexes, there exist two macrocycles in a sandwich-type structure (Figure 8) [269,270,271,272,273,274,275,276,277]; therefore, large pore sizes were expected when these species were trapped inside silica pores. Remarkably, average pore sizes of 2.2 to 2.4 nm were determined for xerogels in which HEu, HSm(TSPc)_2_ or HEu(TSPc)_2_ lanthanides were trapped, respectively. An explanation for these small pore sizes could be the stronger attraction among the eight sulfonic acid (-SO_3_H) groups that are present in the structure of these complexes and the silanol (Si-OH) chains at the pore walls of the growing silica network. Initially, the small size difference between the cavities in which Eu or Sm complexes were trapped was not considered as important; nevertheless, the results of subsequent investigations determined that this difference could be associated to the identity of the lanthanide element.

### 4.2. Physical Trapping or Covalent Bonding of Porphyrins

**Figure 9:**
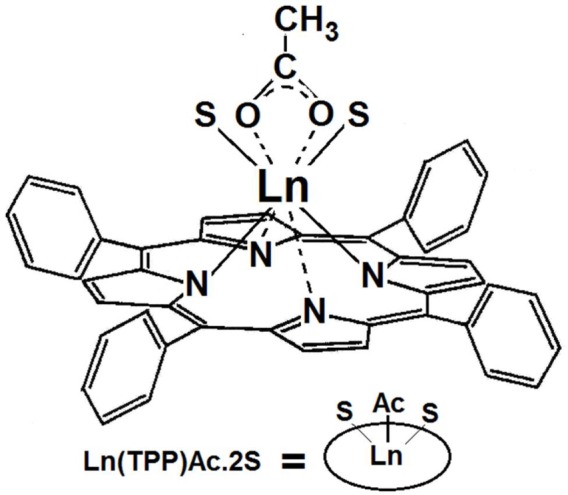
Structure of lanthanide acetate-tetraphenylporphyrins, Ln(TPP)Ac·2S (where Ln = Lu, Yb, Tm, Er, Ho, and S = solvent).

In the next stage of our investigation, the above developed methodology was extended to attempt the encapsulation of porphyrins inside pore networks. As in the case of non-substituted phthalocyanines, many porphyrins are insoluble in polar solvents, such as water or alcohols. Curiously, lanthanide acetate-tetraphenylporphyrins, *i.e*., Ln(TPP)Ac·2S (Figure 9, where Ln = Lu, Yb, Tm, Er and Ho and S = solvent) [437], are soluble in 50% v/v mixtures of ethanol in water. Furthermore, the use of py as a demetallation and protonation inhibiting agent makes possible the entrapping of these species inside the pores of monolithic translucent silica xerogels. In this way, these last compounds were trapped in stable and monomeric forms inside the silica pores.

In the UV-Vis spectra of the Ln(TPP)Ac·2S solution prominent Soret bands and Q_IV_ bands have been observed at 417 nm and 549 nm, respectively, which are characteristic of porphyrinic complexes having a divalent or trivalent cation [42,43,179]. These species can be demetalled and protonated when treated with an HCl solution and then the red-purple solution turns green. As a consequence, the Soret band shifts to 440 nm, while the Q_I_ band shows up at 662 nm, which is due to the presence of H_4_TPP^2+^ dicationic species, in the solution [438,439]. Additionally, the neutral tetraphenylporphyrin free base (H_2_TPP) can be recovered through neutralization via an alkaline solution. The UV-Vis spectra of monomeric free bases of porphyrins are characterized by an intense Soret band at around 420 nm and four bands of decreasing intensities at 515 (Q_IV_), 550 (Q_III_), 590 (Q_II_), and 650 nm (Q_I_).

**Figure 10:**
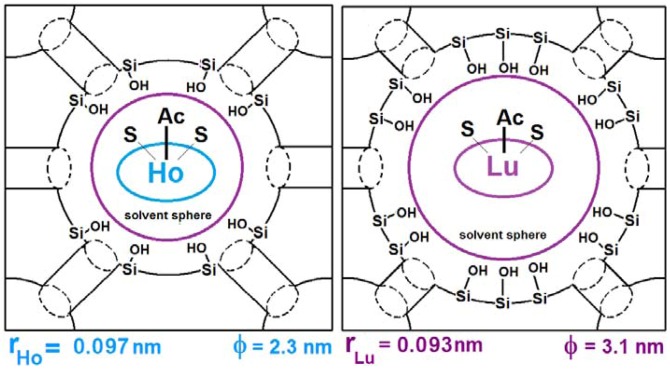
The average pore size of cavities formed around solvated Ln(TPP)As·2S species are a function of the ionic radius of the lanthanide atom that is present in the complex trapped inside the silica network.

In the previous xerogels, the average pore diameters ranged from 2.3 to 3.1 nm (*i.e*., from Ho(TPP)Ac·2S to Lu(TPP)Ac·2S), the size depending on the identity of the lanthanide element present in the respective complex. Apparently, the pore walls are formed around the Ln(TPP)Ac·2S species are surrounded, in turn, by solvation molecules (water, ethanol or siloxane chains), the number of which depends on the size of the lanthanide element entrapped in the porphyrinic complexes [440]. Because of the lanthanide contraction, elements with a higher atomic number (z) are smaller and have smaller ionic radii (r_ionic_), thus attracting more solvation molecules. For this reason, cavities of larger sizes are formed around the Lu complex (z = 71) than those found in samples doped with the Ho complex (z = 67). Even if the ionic radius of Lu^3+^ (r_ionic_ = 0.093 nm) is smaller than that of Ho3+ (r_ionic_ = 0.097 nm), it can still be solvated by more molecules thus causing a larger size cavity. This interesting result was the first confirmation that the size of the cavity formed around a solvated tetrapyrrolic species depends on the identity and structure of the entrapped species (Figure 10).

**Figure 11:**
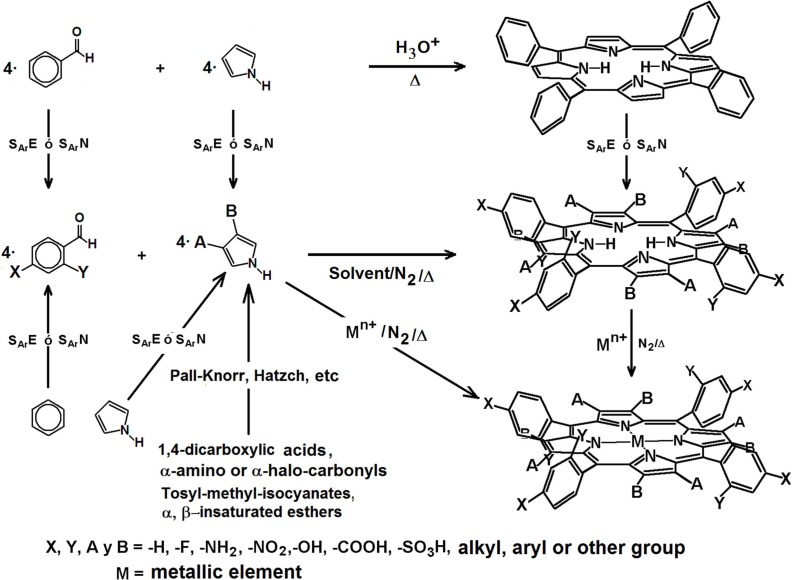
Possible routes to synthesize porphyrins with diverse groups at the A and B positions of pyrroles, or with substituted phenyls attached to the bridge methane groups.

The Ln(TPP)Ac S species were found to be soluble in ethanol and water however, in most cases this would not happen. Depending on the preferred structure, porphyrins can be synthesized by diverse methods. To prepare porphyrins with the desired groups at the pyrrole, A and B positions, it is necessary to bond these groups through electrophilic or nucleophilic aromatic substitutions (SArE, SArN). When these reactions are unfeasible, pyrroles need to be synthesized from precursors such as 1,4-dicarboxylic acids, a-aminocarbonyls, a-halo-carbonyls, a,β-unsaturated esters, *etc*., through the Pall-Knorr or Hatzch reactions, which could be complicated. However, the symmetric tetraphenylporphyrins substituted at the *para* (X) or *ortho* (Y) positions of the phenyls attached to bridging methane groups, H_2_T(X or Y)PP (Figure 11), can be synthesized from pyrrole and substituted benzaldehyde, through the Rothemund reaction (who worked in Fischer’s experiments) [441]. The synthesis and purification of this type of compounds is relatively easy to perform and there exists abundant spectroscopic and physicochemical information about them. The respective complexes of substituted tetraphenylporphyrins can be obtained in one single step from the respective metallic salt, pyrrole and benzaldehyde, or through a two-step method once the porphyrin has been previously synthesized.

To successfully trap, through the sol-gel method, cobalt tetraphenylporphyrins substituted with hydroxyl groups at *ortho* and *para* positions, as well as with amino groups at *para* positions, it was necessary the use of additives such as DMF, methanol, and pyridine to inhibit the dematallation and protonation of the complexes. We have found that the addition of a small quantity of methanol, pyridine, DMF or combinations of them [440], to the gelling mixture, inhibits demetallation and protonation of the macrocycles. Since in *ortho* substituted porphyrins the hydroxyl or amino groups pointing out of the plane of the macrocycle hinder the approach of H_3_O^+^ ions, the entrapping of these species becomes easier. In the case of hydroxyl *para*-substituted porphyrins, a higher amount of pyridine was necessary to inhibit demetallation and protonation; this was possibly due to the strong electroatracting effect of hydroxyl groups over the center of the molecule. The addition of methanol has no effect on the final morphology of the matrix, and the gels formed possess a large number of free vicinal silanol (Si-OH) groups, as it was proved by near infrared spectroscopy (NIR). Also, at relatively low temperatures it is necessary to get rid of all remaining solvents in order to form rigid, optically transparent, and yellowish monoliths. The technique described above has been successfully extended to trap other free and metallic porphyrinic systems.

**Figure 12:**
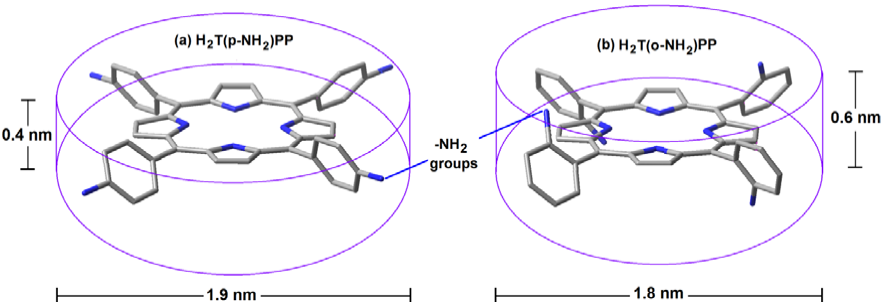
Schematic structures showing the approximate dimensions of isomeric tetraphenylporphyrine substituted with amino groups in: (**a**) *para* or (**b**) *ortho* positions. For simplicity, H atoms and double bonds are omitted.

As it was previously mentioned, many porphyrins show fluorescence in the red region of the spectrum when irradiated with UV light [42,43,179]. Furthermore, many free bases of porphyrins have been proved as possible photosensitizers in PDT, and perhaps through the abovementioned methodology it might be possible to obtain systems suitable to be used in this emerging medical area. For this reason, attempts have been made to trap fluorescent tetraphenylporphyrin free bases substituted at *ortho* (X) or *para* (Y) phenyl positions, H_2_T(X or Y)PP (where *X* = -OH and *Y = *-OH and -NH_2_*)*. In the resultant xerogels, the average pore sizes vary from 2.0 to 3.5 nm, depending on the identity and position of the peripheral substituents [442,443]. As it was previously demonstrated, the smaller sizes were obtained for the cases of entrapping porphyrins having substituting groups at the *ortho*-position, while larger pore sizes were obtained for substituents placed at the *para*-positions. The reason for this difference is that the *para*-substituents are located on the same plane of the molecule while the *ortho*-substitutents are placed at positions pointing up and down from the tetraphenyl-porphyrin molecular plane (Figure 12b). Thus, pore wall formation can be limited by the interactions between these substituents and the growing siloxane chains. Again, these results showed that the sizes of cavities formed around solvated macrocycles are determined by the structure of the trapped species.

To enclose H_2_T(X or Y)PP molecules in monomeric and stable form, the addition of small quantities of DMF, py, MeOH, or combinations of them was required, to act as protonation inhibiting agents. The type or quantity of these additives depended on the substituent groups present in the H_2_T(X or Y)PP species. It was observed that for *para*-substituted molecules a larger quantity of protonation inhibiting agents was required than in the case of *ortho*-substituted species. 

### 4.3. Development and Optimization of Methodologies for Trapping or Bonding Porphyrins and Phthalocyanines within Pore Networks

**Figure 13:**
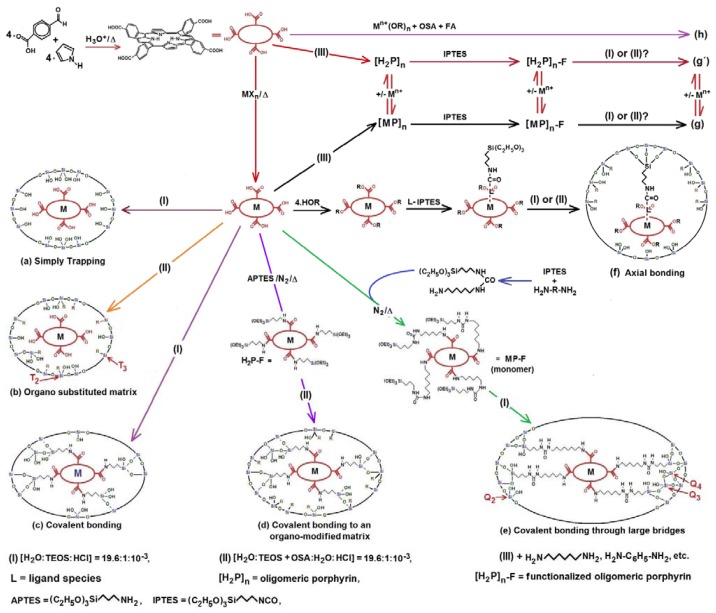
Routes suggested for encapsulating tetrapyrrole macrocycles (using the species H_2_T(P-COOH)PP), as example within porous silica by: (**a**) physical trapping, (**b**) trapping inside an organo-modified matrix, (**c**) covalent bonding, (**d**) covalent bonding inside an organo-modified matrix, (**e**) expansion of the pore cavity by means of attached *diamines* and functionalized alkoxides, (**f**) covalent bonding to the network through a functionalized axial ligand, (**g**) covalent bonding of oligomeric species, (**i**) trapping or bonding in different inorganic matrixes. T_2_, T_3_, Q_2_, Q_3,_ and Q_4_, represent silicon groups detected by ^29^Si-NMR.

The fluorescence of trapped species inside pore networks disappeared or was strongly affected because of its interaction with the SiOH groups on the pore walls. Just in the case of H_2_T(o-NH_2_)PP, the fluorescence observed in the resultant xerogels was similar to that displayed by the free porphyrin in solution. In order to hinder this harmful phenomenon, the next possibilities arise:

- To place the macrocyclic species far from the SiOH groups by establishing bridges between macrocyclic molecules endowed with FA species and the pore walls (Route c in Figure 13).
- To trap the macrocycle inside a network in which SiOH groups have been exchanged with alkyl or aryl groups proceeding from OSA compounds (Route b in Figure 13).
- To create large unions between the macrocycle and the pore walls by combining FA species with polymer precursors such as lactams, diamines or diacids, *etc*. (Route e in Figure 13) or to fix oligomeric species synthesized from tetrapyrrole macrocycles and polymer precursors, [H_2_P or MP]n, inside the cavities of inorganic networks (route g in Figure 13).
- To fix the macrocycle species to the pore walls via an organo-modified network in which the surface SiOH groups have been exchanged by alkyl or aryl groups (Route d in Figure 13).
- To trap or fix monogenic or oligomeric macrocyclic complexes, through axial ligands, inside inorganic or hybrid networks (Route f in Figure 13).
- To combine the use of both FA and OSA compounds for trapping or bonding tetrapyrrolic species inside pore networks of different metal oxides such as ZrO2, TiO2, Al2O3, *etc*. (Routes g and h in Figure 13).

**Figure 14:**
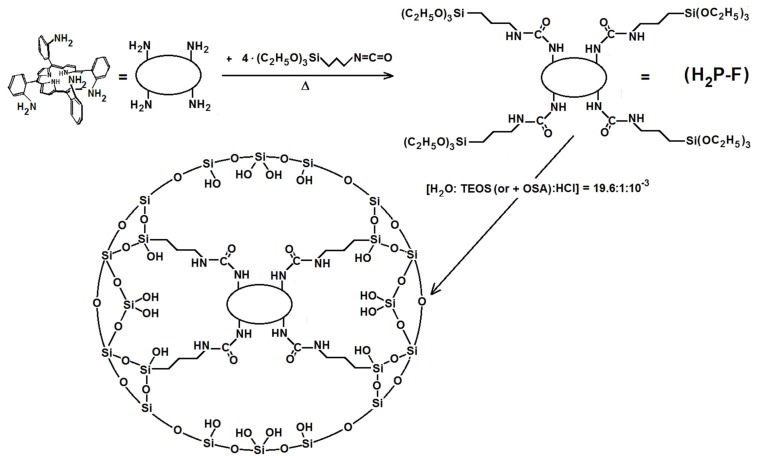
Reaction scheme for bonding of porphyrin free bases, H_2_T(o-NH_2_), to the pore walls through the use of IPTES to form the respective H_2_P-F precursor, and its subsequent entrapping inside the silica pores.

In order to explore the effect of separating macrocycle species from the pore walls, free bases of *X*-substituted porphyrins (where X = o-NH_2_ or Y= p-COOH, p-OH) were bonded to the silica network through the use of functionalized alkoxides (FA), such as isopropyltriethoxysilane (IPTES), 3-aminopropyltriethoxysilane (APTES), *N*-(2-aminoethylamino)propyltrimethoxysilane (NAEPTES), *etc*. The process of formation of a covalent union between FA compounds and X or Y porphyrin groups to render H_2_P-F precursors, was monitored through FTIR spectroscopy [443]. In a second step, the H_2_P-F species was bonded to the network that was generated from the hydrolysis and condensation of TEOS (Figure 14). The UV-Vis spectra showed that in these systems porphyrins remained in monomeric and neutral forms. The comparative fluorescence analyses of species, either simply trapped or covalently bonded to silica, confirmed that fluorescence disappears by interactions of the macrocycles with SiOH groups, while in the second case fluorescence was better preserved [443].

N_2_ sorption on these xerogel substrates can produce IUPAC Type I, Type II or Type IV isotherms [444,445]. The hysteresis loops corresponding to encapsulated macrocycles in SiO_2_ networks are often very narrow, in which the access to and exit from the pore system takes place from an assemblage of cavities enclosing the macrocyclic species, which are interconnected through narrow necks of varying sizes. The pore cavity sizes of the silica substrates can be approximated by the Non-Local Density Functional Theory (NLDFT) [444,445]. In general, we expect to have relatively wide pore cavities interconnected by narrow throats throughout the network in our SiO_2_-macrocycle systems. For simplicity, in the present manuscript, we have employed a simple cylindrical pore model together with the NLDFT approach; nonetheless, the cavity sizes produced by entrapment of macrocycle species may be better calculated, in the future, if an elliptical (in view of the porphyrin and phthalocyanine molecule shape) pore model is considered.

**Figure 15:**
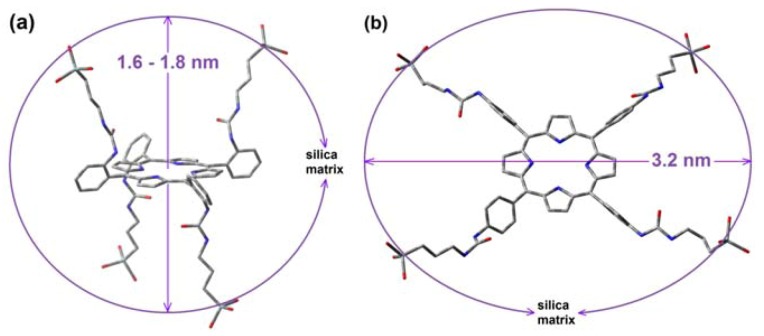
Depending on the position of amino groups in: (**a**) H_2_T(o-NH_2_)PP or (b) H_2_To(p-NH_2_)PP (**b**) species, small or large pore widths, (Φ), can be obtained. For simplicity, hydrogens in these structures have been omitted.

In the case of samples containing substituted free porphyrins chemically bonded to the silica walls, the depicted average widths ranged from 1.6–1.8 to 4.0 nm while the specific surface areas varied from 459 to 632 m^2^/g [446,447,448]. Again, as was observed in the case of the simple trapping of *ortho-* or *para*-substituted porphyrins, the smallest pore sizes occurred in samples prepared with *ortho*-NH_2_ substituted porphyrins whilst the largest pore sizes arose for porphyrins having substituent groups at the *para*-positions (Figure 15a,b). In other words, as a consequence of the dimensions and ellipsoidal-like shape of the trapped porphyrin molecules (see Figure 12), the size and shape of the cavity built around the porphyrin molecule depended upon the structure of the molecule that was being trapped or fixed inside the gel matrix. 

This last observation confirmed the Dunn and Zink assertion [383] that, through the sol-gel process, the nature of the dopant molecule determines the structure of the cavity that is formed around the trapped molecule, as we have proved by using porphyrins. The effect also occurs with species smaller than the protein used by the abovementioned authors, and depends on other subtle factors such as the position and identity of the substituents in the structure of porphyrins, and the identity of the cation in the respective complexes. Recently, Peng and Lu found [449] structures such as the H_2_P-F precursors, herein described (Figure 14) that make possible the synthesis of mesoporous and functional nanocomposites without the use of surfactants. As occurs in our systems, the presence of tetrapyrrolic species, such as porphyrins (even if related species could also be used), induce the directional assemblage of the network structure.

**Figure 16:**
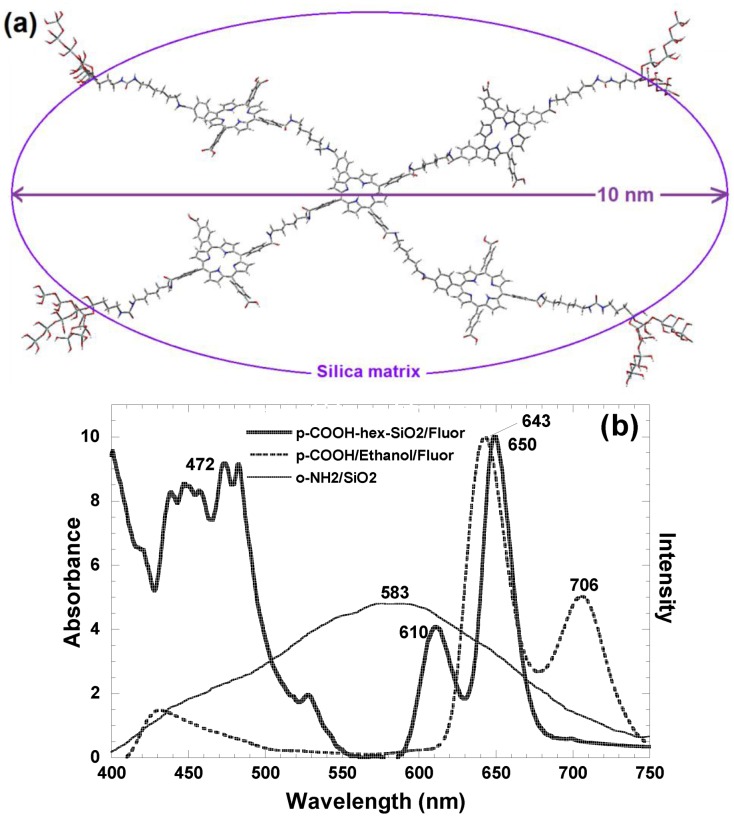
(**a**) Average pore sizes extending from 4.4 to 10 nm can be obtained when the H_2_P-F precursor proceeds from the reaction of H_2_(p-COOH)PP, hexanodiamine, and IPTES. (**b**) In these systems, the fluorescence of H_2_(p-COOH)PP species is similar to that displayed by this same species in solution or when H_2_T(o-NH_2_)PP molecules are simply trapped inside silica.

As was previously observed, the bonding of porphyrins to the pore walls by means of only using FA species as bridges was not enough to inhibit the effects of Si-OH groups on the fluorescence and reactivity of porphyrins; therefore, the possibility of enlarging these unions was explored. In order to do this, polymer precursory species such as 1,6-hexanodiamine were initially bonded to IPTES and subsequently to H_2_(p-COOH)PP then yielding long branched precursors, H_2_P-F, which afterward reacted with TEOS and H_2_O to create the pore network around the porphyrin (Route e in Figure 13). This two-step process was monitored by FTIR spectroscopy and the resultant translucent monolithic xerogels showed fluorescence similar to that observed in free porphyrin solutions. The pore sizes ranged from 4.4 to 9.4 nm (Figure 16a) and the only explanation for having these relative large pore sizes was the formation of longer bridges with the pore walls containing two or more macrocyclic molecules per void. These results confirmed the possibility of enlarging and modulating the pore size of silica xerogels through the use of tetraphyrrolic species; nevertheless, it is still possible the use of other chemical species for this pore templating task.

The possibility of preserving the reactivity, spectroscopic, and luminescent properties of tetraphyrrolic species trapped inside silica pores could be initially attempted through the exchange of Si-OH groups with alkyl or aryl groups. This option was explored by using the (OH)AlTSPc species once more as a probe (Route b, Figure 13). In this way, it was found that translucent and monolithic xerogels could be obtained when using as low as 2% v/V_f_ of assorted OSA compounds, such as: dimethyldiethoxysilane, (dMeTEOS), amyltriethoxysilane (AmTEOS), vinyltriethoxysilane (VyTEOS), ethyl-triethoxysilane (EtTEOS), allyltriethoxysilane (AlyTEOS), and dodecyltriethoxysilane (DoTEOS). Spectroscopic analyses revealed that (OH)AlTSPc probe species remained in monomeric and stable form when ethyl or amyl groups were attached to the surface of the pore network [450]. Possibly, the polarity induced by the close presence of ethyl or amyl groups to the macrocyclic molecules made easier the occurrence of electronic transitions in the trapped (OH)AlTSPc species.

In these samples, the average pore sizes varied from 1.6 to 3.8 nm and depended on the nature of the alkyl groups attached to the pore walls. The smallest pore sizes were obtained when the attached alkyl groups were ethyl or amyl, while the largest sizes were achieved when these groups were methyl or vinyl. Apparently, small sizes were obtained due to attractive interactions of macrocycles with ethyl and amyl groups while large sizes were obtained because of the repulsive interactions of the phthalocyanine toward methyl or vinyl groups attached to the pore walls. Besides, the specific surface area result a function of the identity of the alkyl group attached to the silica surface and varied from 688 to 841 m^2^/g. These values were higher than the 540–631 m^2^/g values determined for pristine silica xerogels [451]; these results showed the effect of the presence of organic groups over the textural properties of the network. The last results suggested the possibility of modulating pore sizes, even though the most important effect is the possibility of tuning the polarity inside the silica cavities by exchanging Si-OH groups with alkyl or aryl groups.

**Figure 17:**
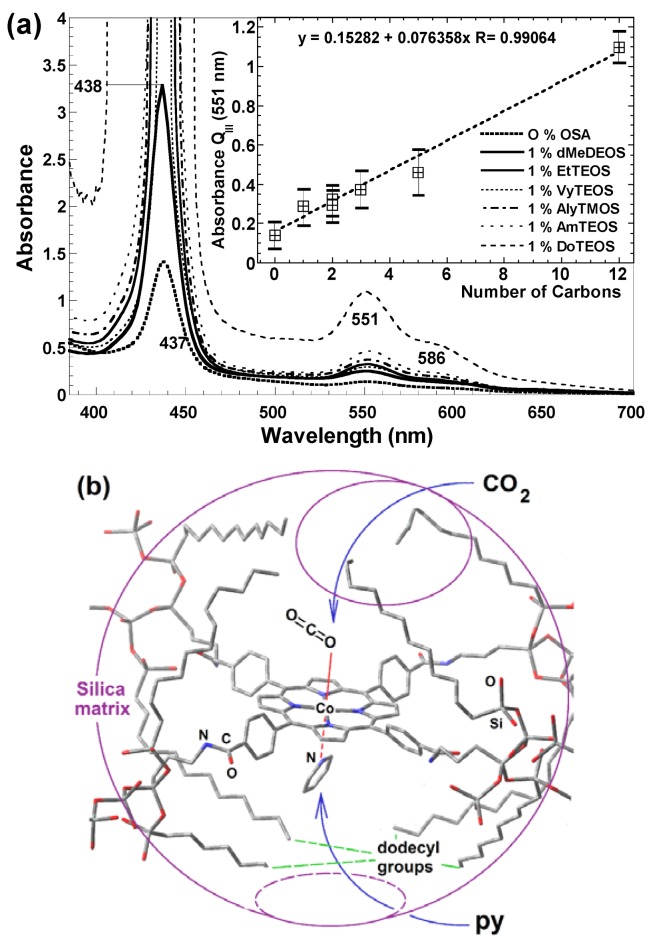
(**a**) In the UV-Vis spectra of CoT(p-COOH)PP bonded to the pore walls of SiO_2_ organically modified viaalkyl groups the O_III_ band at 551 nm, apparently depends on the number of carbons of the alkyl groups attached to the silica network. (**b**) Hypothetical situation of a Co porphyrin inside a pore cavity.

Consequently, the next obvious step of our investigation was the preparation of translucent monolithic xerogels in which a tetrapyrrolic species, such as CoT(p-COOH)PP, bonded to organo-modified silica. The UV-Vis spectra of this sample (Figure 17a) revealed that the Co complex remained bonded and stable inside the silica pores. However, in these xerogels, the absorbance depicted by the respective UV-Vis spectra was proportional to the number of carbons present in the alkyl group attached to the pore surface [452]. Apparently, the alkyl chains remained close of the porphyrin molecule thus inhibiting the approach of interfering polar species. Large alkyl chains induce a lower polar environment inside the pores, then making easy the occurrence of UV-Vis transitions of porphyrin (Figure 17a). In these systems, the average pore sizes (F) ranged from 2.9 to 3.3 nm and the specific surface areas were function of the length of alkyl groups. Chains including double bonds, such as vinyl or allyl groups, rendered smaller pore sizes while longer chains, such as amyl or dodecyl, induced the formation of larger pore sizes with lower polarities. Thus, the last results confirmed that the presence of alkyl groups helped to modulate the polarity inside the pores and facilitated the display of spectroscopic, luminescent and coordination properties of porphyrins that were bonded to the pore network.

Furthermore, the understanding and optimization of the above described methodology made possible the design of cavities with functional species inside, which could be applied in optical and luminescent devices, for the selective absorption of diverse gases (Figure 17b), toxic substances or to create original catalytic systems.

### 4.4. Research in Progress

Recently, our research group has continued studies related to the synthesis of systems based on the bonding of oligomeric porphyrins for producing larger pore sizes. Furthermore, we are exploring the possibility of trapping or bonding tetrapyrrolic species inside the pores of ZrO2 xerogels synthesized by the sol-gel method. The first result, explored with MTSPc species, suggests that macrocyclic species can be trapped in stable and monomeric forms inside cavities having low amounts of interfering groups (*i.e*., Si-OH groups of silica) since the higher reactivity of Zr alkoxides produces a highly intricate network with fewer ramifications. Apparently, there arises a lower polarity in the pores of Zr xerogels than that existing in silica. This lower polarity provokes the MTSPc species to remain in stable and disaggregated forms while strongly displaying their electronic transitions. The average pore diameters determined in these xerogels ranged from 1.9 to 2.0 nm, depending on the identity of the cation in the MTSPc species (M = Fe, Co, Ni, Cu or Al). However, the exploration of the latter methodology is still under way at this moment. Furthermore, recently the methodology developed to trap MTSPc was applied to chemically bond species, such as CoT(p-COO)PP or [H_2_T(CO_2_H)PP]_5_ oligomers. With the CoT(p-COO)PP species, average pore diameters of about 3,4 nm were determined, and large sizes of around 10.7 nm were obtained when oligomeric species were bonded to the ZrO2 matrix. These results confirm again that the structure of the macrocyclic species determine the sizes of pores or even their shapes.

As shown in the scheme of Figure 13, there still exists the possibility of combining some of the developed methodologies to improve the characteristics of the final systems and to take advantage of the transcendental properties of tetrapyrrolic macrocycles. At present, our research group explores the creation of polymeric matrices containing tetrapyrrolic compounds as integral part of the structure. Furthermore, this methodology is now being used to try fixing natural tetrapyrrols, such as native chlorophyll a and heme groups, and to use the resultant solid systems as sensors, catalysts or medical devices.

Finally, all the abovementioned results strongly hint at the possibility of controlling the pore size, shape, specific surface area, and also to tune the polarity inside the pores of a network by choosing the appropriate OSA and/or FA alkoxides together with pertinent tetrapyrrolic macrocyclic molecules employed as templating species. As it can be seen in Table 1, such properties can be modulated through the adequate choice of the structure of tetrapyrrolic species and of pore network precursors. Thus, an option for the appropriate design of cavities or pore engineering [383,393], to hold guest molecules may be developed by taking into account the above described results and methodologies. Nevertheless, we think that the development of alternative methodologies can lead to the efficient trapping of diverse organic, biochemical, organo-metallic, and metallorganic species. In this way, the generated systems could involve important scientific and technological applications.

Perhaps the tremendous efforts that have been made to elucidate the structure and properties of tetrapyrrole macrocycles could now lead to unsuspected consequences. The most unexpected result among all above is that the structural implications and properties of tetrapyrrolic macrocyclic molecules may be used to optimize and create totally synthetic devices, which facilitate the solution of some of the current and most dangerous problems related to mankind.

**Table 1:** Textural properties, evaluated by N2 adsorption, of xerogels with diverse tetrapyrrolic macrocyclic species trapped or bonded to the pore network. The blank is a specimen synthesized from TEOS without including trapped or bonded macrocyclic species.

Trapped species	Entrapment	Network	Average pore size φ/nm	Surface m^2^/g
Blank	physically trapped	pristine SiO_2_	3.4	729
(OH)AlTSPc	physically trapped	SiO_2_	2.7	585
MTSPc (M = Fe, Co, Ni, Cu, Al)	physically trapped	SiO_2_	2.7–3.5	540–631
HLn(TSPc)_2_ (Ln = Eu and Sm)	physically trapped or covalently bonded	SiO_2_	2.2–2.4	
Ln(TPP)Ac·2S (Ln = Ho to Lu)	physically trapped	SiO_2_	2.6–3.1	
H_2_T(X or Y)PP (*X = OH or Y = OH, NH_2_*)	physically trapped	SiO_2_	2.0–3.5	
H_2_T(o-*X*)PP	covalently bonded	SiO_2_	1.6–1.8	459–632
H_2_T(p-*Y*)PP	covalently bonded	SiO_2_	3.2–4.0	459–632
[H_2_T(*X or Y*)PP]n (oligomers)	covalent bonded	SiO_2_	4.4–9.4	463–610
(OH)AlTSPc	physically trapped	organo modified-SiO_2_	1.6–3.8	688–841
CoT(p-COOH)PP	covalently bonded	organo modified-SiO_2_	2.9–3.5	410–621
MTSPc (M = Fe, Co, Ni, Cu, Al)	physically trapped	ZrO2	1.9–2.0	

Where, MTSPc = Tetrasulfophthalocyanine, TPP = tetraphenylporphyrin, Ln = a lanthanide element, H_2_T(X or Y)PP = free bases of substituted tetraphenylporphyrins. Data from references [431,432,433,434,435,436,437,438,439,437,440,442,443,446,447,448,449,450,451,452].

## 5. Conclusions

The methodologies employed to physically trapping or covalently bonding tetrapyrrolic species in xerogel pore networks were developed using (OH)AlTSPc and H_2_T(X or Y)PP species as probes, in order to find the optimal mixtures that can render translucent and monolithic either pristine or organo-modified silica xerogels. The spectroscopic analysis of the trapped probe species reveals that their interactions with the Si-OH surface groups on the pore walls affect their stability and physicochemical properties. In order to inhibit these negative effects, strategies such as: (i) locating these compounds far from the pore walls, (ii) exchanging Si-OH groups or (iii) trapping oligomeric macrocycles have been proposed and explored. The results obtained suggest that the size, possibly the shape and the internal physicochemical environment of the pore network formed around the solvated macrocycles, mainly depend on the structural characteristics of these species. Through the use of the methodologies herein described, it was possible to trap in monomeric and stable forms macrocyclic species inside the pores of a solid substrate; the spectroscopic and luminescent properties of which become similar to those observed in solution. Furthermore, the macrocyclic species can be chemically bonded to the pore walls of a silica network, while the polarity inside the pores can be tuned through the substitution of Si-OH surface groups by alkyl or aryl chains proceeding from the respective OSA compounds. In this this way, the spectroscopic and fluorescence properties of the macrocyclic species can be optimized by choosing the adequate alkyl or aryl group.

As shown above, the properties of tetrapyrrolic macrocycles can be improved through their trapping inside inorganic, hybrid or polymeric networks. However, the most important aspect of this research is that the structural versatility of porphyrins, phthalocyanines, *etc*., can be related to chlorophyll and heme group and can help in the design of new types of modern materials.

The methodology described herein strongly suggests the possibility of achieving an authentic design and engineering of pore systems suitable for use in catalytic, optical, sensor or medical devices**. **Furthermore, recent development of methodologies and the application of the sol-gel process for the synthesis of pristine metal oxides or mixtures of these oxides, mesoporous networks, composites, thin films, optical quality xerogels, aerogels, biosensors, nanostructures, nanoparticles, and core/shell nanoparticles, have caused a surge in new interdisciplinary research areas. The combination of these methodologies and the use of tetrapyrrole macrocycles could lead to the creation of novel materials suitable to be used in strategic technological areas. The sense of the present review was to show that in the structure and characteristics of tetrapyrrole macrocycles remain immersed some of the most transcendental secrets of life in our planet and that the investigations of their use will be necessarily impregnated with its beauty.
